# Towards Recycling of All‐Solid‐State Batteries with Argyrodite Sulfide Electrolytes: Insights into Electrolyte and Electrode Degradation in Dissolution‐Based Separation Processes

**DOI:** 10.1002/cssc.202402128

**Published:** 2025-01-20

**Authors:** Kerstin Wissel, Zian Hu, Xuebin Wu, Martine Jacob, Kathrin Küster, Ulrich Starke, Oliver Clemens

**Affiliations:** ^1^ Institute for Materials Science Chemical Materials Synthesis University of Stuttgart Heisenbergstraβe 3 70569 Stuttgart Germany; ^2^ Max Planck Institute for Solid State Research Heisenbergstraße 1 70569 Stuttgart Germany; ^3^ Institute for Materials Science Materials Analysis Technical University of Darmstadt Alarich-Weiss-Straße 2 64287 Darmstadt Germany

**Keywords:** Thiophosphate, Argyrodite, Recycling, Li_6_PS_5_Cl, Li_6_PS_5_Br, Li_6_PS_5_I, All-solid-state batteries

## Abstract

All‐solid‐state Li‐ion batteries (ASSBs) represent a promising leap forward in battery technology, rapidly advancing in development. Among the various solid electrolytes, argyrodite thiophosphates Li_6_PS_5_X (X=Cl, Br, I) stand out due to their high ionic conductivity, structural flexibility, and compatibility with a range of electrode materials, making them ideal candidates for efficient and scalable battery applications. However, despite significant performance advancements, the sustainability and recycling of ASSBs remain underexplored, posing a critical challenge for achieving efficient circular processes. This study investigates the dissolution‐based separation and recovery of argyrodite thiophosphate electrolytes and transition metal oxide electrode materials as a potential recycling strategy for ASSBs. A focus is set on the impact of solvent treatments on the recrystallization behavior of these electrolytes. Furthermore, the interactions between dissolved argyrodite thiophosphates and various transition metal oxide electrode materials (LiCoO_2_, LiMn_2_O_4_, LiNi_0.8_Mn_0.1_Co_0.1_O_2_, LiFePO_4_ and Li_4_Ti_5_O_12_) is examined to assess their influence on the functional properties of both the electrolytes and electrode materials. Structural, compositional and morphological changes are analyzed using X‐ray diffraction, scanning electron microscopy, energy‐dispersive X‐ray spectroscopy, inductively coupled plasma mass spectrometry and X‐ray photoelectron spectroscopy. Our findings provide insights into the complexities of recycling ASSBs, but also highlight the potential for developing efficient, sustainable recycling processes.

## Graphical abstract

This study explores the complexities and potential of recycling all‐solid‐state Li‐ion batteries based on argyrodite sulfide solid electrolytes through a dissolution‐based separation strategy. It examines the impact of solvent selection on electrolyte recrystallization and the effects of interactions between electrolyte and electrode materials on the functional properties.

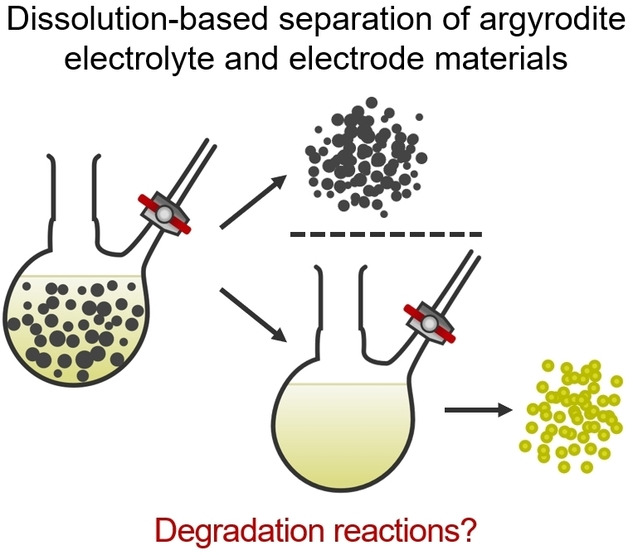

## Introduction

All‐solid‐state Li‐ion batteries (ASSBs) are promising next‐generation batteries due to their high energy densities and improved safety.[^1^, ^2^, ^3^] In the search for suitable solid electrolytes (SEs), a multitude of materials including oxide‐, polymer and sulfide‐based electrolytes has been extensively investigated. Sulfide‐based electrolytes, particularly argyrodite thiophosphates Li_6_PS_5_X (X=Cl, Br, I), have garnered significant interest due to their high ionic conductivities. Argyrodites were initially introduced by Deiseroth et al.^[4]^ in 2008 as fast lithium‐ion conductors, exhibiting ionic conductivities up to 10^−2^ S ⋅ cm^−1^. The differences in the crystal structure (i. e., structural disorder) due to different sizes of the halide anions in Li_6_PS_5_X (X=Cl, Br, I) influence the ionic conductivities of the SEs significantly.[^4^, ^5^] While Li_6_PS_5_Cl and Li_6_PS_5_Br are known to have comparatively high ionic conductivities, a lower ionic conductivity in the order of 10^−6^ S ⋅ cm^−1^ is observed for Li_6_PS_5_I.[^6^, ^7^]

Several synthesis methods have been employed to produce Li_6_PS_5_X (X=Cl, Br, I), covering a broad spectrum from solid‐state synthesis[^4^, ^8^], mechanochemical milling[^9^, ^10^] to solution‐based synthesis methods.[^11^, ^12^, ^13^, ^14^, ^15^, ^16^] Solution‐based synthesis approaches, classified into suspension and dissolution‐precipitation methods, are considered more efficient and scalable, sparking significant research interest in their development.[^17^, ^18^] Furthermore, sulfides can be processed in various solvents. A distinction can be made between slurry processing (involving suspensions of SE and other components) and solution processing (again dissolution‐precipitation of SE). These post‐processing steps can be utilized, for example, in the fabrication of composite electrodes, coating of active electrode materials, and production of electrolyte films.[^17^, ^18^]

**Figure 1:**
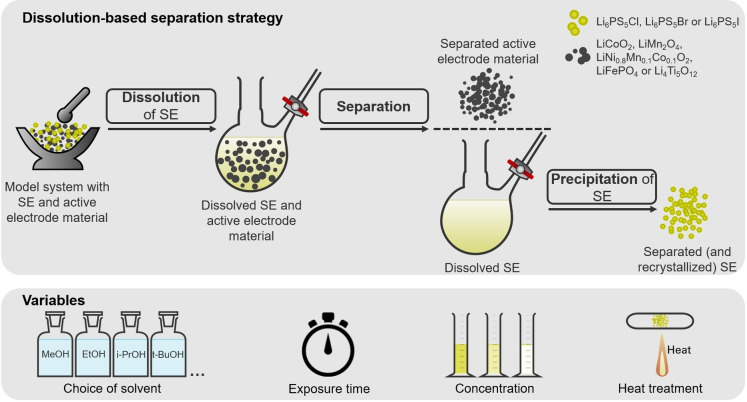
Diagram of dissolution‐based separation process, highlighting key variables explored, including choice of solvent, exposure time, concentration, and potential heat treatment required for recrystallization of SE.

With the emergence and growing significance of ASSBs, it is crucial to recognize that many of the recycling strategies employed for conventional Li‐ion batteries (LIBs) may not be directly applicable to ASSBs. For conventional lithium‐ion batteries, various approaches have been established, including pyrometallurgical, hydrometallurgical, and direct recycling methods.[^19^, ^20^, ^21^, ^22^] These methods often focus solely on recovering valuable critical elements or regenerating active cathode materials.[^23^, ^24^] Due to the relatively recent introduction of ASSBs with many different battery chemistries, developing a universal recycling approach is challenging. However, this also presents an excellent opportunity to proactively plan and design recycling processes to establish a sustainable system for ASSBs.^[25]^ So far, only a few studies have been focused on recycling of ASSBs.^[26]^ Compared to conventional LIBs, recycling ASSBs presents additional challenges due to the more complex material mix. This includes not only active electrode materials, binders, and other components but also valuable solid electrolytes, which add significant production complexity and value. As a result, the recycling process becomes significantly more complex. Consequently, direct recycling strategies allowing for an efficient separation and recovery of individual components are important. Given the potential for solution processing of argyrodite thiophosphates electrolytes, a promising approach for achieving separation of the electrolyte from other insoluble battery components involves dissolution and subsequent precipitation through solvent removal and recrystallization of the SE.^[25]^ The corresponding process is illustrated in Figure 1. Pyrometallurgical and hydrometallurgical processes, on the other hand, are impractical because reactions with water or O_2_ degrade the thiophosphate units of the SEs.^[26]^

A first proof‐of‐principle of this dissolution‐based separation strategy has been reported by Tan et al.^[27]^ for a model system of Li|Li_6_PS_5_Cl|LiCoO_2_ using EtOH as the solvent. Though they could show that separation and recovery/regeneration of the electrolyte and electrode material are possible by employing this strategy, a detailed investigation on the impact of the solvent treatment, in particular with regards to the choice of organic solvent, the concentration of the electrolyte in the solvent as well as of the exposure time, has not been performed yet. In addition, so far, no attention has been given to the influence of the presence of other electrode materials during the dissolution process. In a recently published study,^[28]^ we investigated the interactions between different commonly used transition metal oxide electrode materials and dissolved β‐Li_3_PS_4_. We found that the presence of electrode materials in the dissolution process can lead to significant chemical reactions. These interactions can lead to substantial changes in the functional properties of individual components. A strong dependence on the electrode materials used was found and different degradation reactions were observed. This is in agreement with the fact that thiophosphates possess a narrow electrochemical stability window, which makes them unstable towards charged electrode materials and leads to electrolyte degradation.^[29]^ In addition, it should be noted that electrode materials contain transition metal species with oxidation states higher than their preferred states within binary oxides or sulfides, even in their discharged state, promoting redox reactions when the SEs are dissolved.^[26]^ In solution, argyrodite electrolytes decompose into their ionic components according to the equation:

$${Li}_{6}PS_{5}X\;\left(X=Cl,\;Br,\;I\right)\to 6{Li}^{+}+{PS}_{4}^{3-}+S^{2-}+X^{-}.$$

Thus, in contrast to β‐Li_3_PS_4_ which only produces PS_4_^3−^ anions (and possibly minor traces of S^2−^) during dissolution, Li_6_PS_5_Cl, Li_6_PS_5_Br and Li_6_PS_5_I form additional Cl^−^, Br^−^ and I^−^ as well as S^2−^ anions. These two additionally solvated ionic species X^−^ and S^2−^ can interact with the electrode materials at their surfaces. In general, the presence of multiple anions can lead to additional interactions and competing reactions can occur, further increasing the complexity of the chemistry. Several aspects must be considered, including the occurrence of redox reactions, factors influencing complex formation and stability, solubility and precipitation, etc. According to the standard potentials of these anions, they are ordered by their reductive strengths as follows: S^2−^ > I^−^ > Br^−^> Cl^−^. Based on the electrochemical series, cations such as Mn^4+^, Mn^3+^, Fe^3+^, Co^3+^ and Ni^3+^ are unstable against S^2−^ and (partially) against I^−^. Furthermore, the solubility chemistry of transition metal sulfides and halides is markedly different. While almost all divalent cations of Mn, Fe, Co and Ni form sulfides with low solubility, their corresponding halides have typically high solubilities. Therefore, sulfide ions in solution can particularly lead to the precipitation of transition metal sulfides. Overall, these interactions might lead to significant electrolyte and electrode deterioration. This study aims to investigate the nature of possible interactions between dissolved argyrodite thiophosphates and different transition metal oxide electrode materials (layered‐type LiCoO_2_ (LCO) and LiNi_0.8_Mn_0.1_Co_0.1_O_2_ (NMC811), spinel‐type LiMn_2_O_4_ (LMO) and Li_4_Ti_5_O_12_ (LTO), and olivine‐type LiFePO_4_ (LFP)), and their influence on functional properties of both the electrolytes and the electrode materials. Before exploring this, a focus is set on the examination of the influence of solvent treatments o the electrolytes, their concentrations in the solvents and the duration of the dissolution process on the recrystallization behavior of the electrolytes. The structural changes in the argyrodite electrolytes and electrode materials are analyzed using X‐ray diffraction (XRD) combined with Rietveld analysis. Morphological and compositional alterations are investigated through scanning electron microscopy (SEM), energy‐dispersive X‐ray spectroscopy (EDX), inductively coupled plasma mass spectrometry (ICP‐MS) and X‐ray photoelectron spectroscopy (XPS). Additionally, the ionic conductivity of the recycled argyrodite electrolytes and the electrochemical performance of the recycled electrode materials are evaluated. These analyses provide valuable insights into the compatibility and effectiveness of the solution‐based recycling process for different battery components.

## Experimental

### Material Preparation

Pristine Li_6_PS_5_Br and Li_6_PS_5_I were prepared via a mechanochemical milling route followed by an annealing step, adapted from literature[^10^, ^30^, ^31^, ^32^]. For this, the precursor materials Li_2_S (Alfa Aesar, 99.9 %) and P_2_S_5_ (Sigma‐Aldrich, 99 %) were mixed with LiBr (Sigma‐Aldrich, ≥99 %) or LiI (Sigma‐Aldrich, 99.9 %) in a stoichiometric ratio of n(Li_2_S):n(P_2_S_5_):n(LiBr/LiI)=5 : 1 : 2. A typical batch size of 5 g was targeted. A pre‐mixing process was carried out using mortar and pestle. The mixture was then ball‐milled inside an air‐tight 50 ml ZrO_2_ jar with 10 ZrO_2_ balls with a diameter of 10 mm at 550 rpm for 16 hours under Ar atmosphere. After ball milling, the obtained powder was transferred into quartz ampoules and sealed under vacuum (p ≈1 ⋅10^−3^ mbar). The sealed ampoules were subjected to a heat treatment at 550 °C for 10 hours. Pristine Li_6_PS_5_Cl was purchased from NEI Corporation (USA). All synthesis steps, materials storage as well as further material handling were carried out in an Ar‐filled glovebox with oxygen and water levels below 1 ppm, respectively, or in vacuum.

Pristine electrode materials LiCoO_2_ (LCO), LiMn_2_O_4_ (LMO), LiNi_0.8_Mn_0.1_Co_0.1_O_2_ (NMC811) and Li_4_Ti_5_O_12_ (LTO) were synthesized by solid‐state reactions[^33^, ^34^, ^35^, ^36^] using Li_2_CO_3_ (AlfaAesar, 99 %), Co_3_O_4_ (Thermo Scientific, 99.7 %), MnO (AlfaAesar, 99 %), Mn_2_O_3_ (Sigma‐Aldrich, −325 mesh, 99 %), NiO (Sigma‐Aldrich, <50 nm, 99.8 %) and TiO_2_ (Thermo Scientific, anatase, −325 mesh, 99.6 %) as precursor materials. Mixtures of stoichiometric amounts of the respective oxides were prepared by intimately grinding using mortar and pestle and heated to 850 °C for 12 h in air, before regrinding and reheating at the same temperature for further 12 hours. An excess of 5 wt.% Li_2_CO_3_ was used to compensate Li losses during the heat treatments. LiFePO_4_ (LFP) was prepared according to literature.^[37]^ For this, stoichiometric amounts of Li_2_CO_3_ (Alfa Aesar, 99 %), FeC_2_O_4_ ⋅ 2H_2_O (Sigma‐Aldrich, 99 %) and NH_4_H_2_PO_4_ (Alfa Aesar, 98 %) were ball‐milled at 300 rpm for 3 hours. Some isopropanol (i‐PrOH, VWR, ≥99.7 %) was added to facilitate better mixing and homogenization of the powders. Afterwards a two‐step heating treatment was applied: First, the mixture was heated at 350 °C for 10 hours. After an intermediate grinding step, the powder was subjected to a second heating process at 600 °C for 10 hours. Both heat treatments were conducted under Ar atmosphere. It is important to note that this synthesis approach produces non‐carbon‐coated LFP. The absence of a carbon coating on the LFP enables to isolate and directly attribute any changes in electrochemical activity to reactions between LFP and the dissolved electrolyte, eliminating the influence of a carbon coating as an additional variable. All freshly prepared electrode materials were directly transferred into an Ar‐filled glovebox.

### Dissolution and Recrystallization of Argyrodite‐Type Electrolytes

For the dissolution of the electrolytes Li_6_PS_5_Cl, Li_6_PS_5_Br, or Li_6_PS_5_I, ethanol (EtOH, AlfarAesar, max 0.003 % H_2_O) was used as solvent. For Li_6_PS_5_Cl, a detailed study was conducted investigating the influence of the concentration of the electrolyte in EtOH as well as of the reaction time on the recovered products. Various solid:liquid ratios (200 mg:1 ml, 100 mg:1 ml and 10 mg:1 ml) and varying stirring lengths (1 h vs. 24 h) were examined. Additionally, the dissolution behavior of Li_6_PS_5_Cl in other solvents including methanol (MeOH, anhydrous, 99.9 %, thermo scientific), isopropanol (i‐PrOH, anhydrous, max. water 0.003 %, VWR Chemicals), tert‐Butyl alcohol (tert‐BuOH, anhydrous, ≥99.5 %, Sigma‐Aldrich), hexane (anhydrous, Alfa Aesar), tetrahydrofuran (THF, anhydrous, 99.8+%, Alfa Aesar), ethyl acetate (EA, 99.8 %, Sigma Aldrich), acetonitrile (ACN, anhydrous, 99.8+ %, Alfa Aesar), and N‐methyl formamide (NMF, 99 %, thermo scientific) was assessed. For Li_6_PS_5_Br and Li_6_PS_5_I, experiments were conducted using EtOH as solvent with the lowest concentration of 10 mg:1 ml and stirring durations of 24 h. Physical and chemical properties of solvents used are listed in Table S 1. Karl Fischer titration (Titrator Compact C10SX, Mettler‐Toledo) was used to verify the residual water content of the solvents. If the solvents were not purchased in anhydrous form, they were dried over molecular sieves (3 Å, 20 % m/v, Sigma‐Aldrich).

To remove the solvents, the Schlenk‐flask filled with the solutions was connected to a Schlenk line for distillation and heated to 120 °C for 2 hours under vacuum (p ≈5–8 ⋅ 10^−2^ mbar). Prior to sealing the samples in quartz ampoules under vacuum (p ≈1 ⋅ 10^−3^ mbar), any remaining solvent was removed via an additional heating step at 300 °C for 2 h under vacuum (p ≈5–8 ⋅ 10^−2^ mbar). To increase the degree of crystallinity of the precipitated powder, the sealed ampoules were heated at 550 °C for 10 h. For samples treated with hexane, THF, EA and NMF, the heating step to 550 °C was not performed.

### Separation of Argyrodite‐Type Electrolytes from Electrode Materials

Pristine Li_6_PS_5_Cl, Li_6_PS_5_Br or Li_6_PS_5_I and the electrode materials (resulting in 15 different material combinations consisting of one electrolyte and one electrode material each) were mixed using mortar and pestle in a 1 : 1 weight ratio. Subsequently, EtOH was added using a solid:liquid ratio of 10 mg:1 ml. After 24 h of stirring, undissolved precipitates were separated from the solution using centrifugation (6000 RPM (corresponding to a relative centrifugal force of 3622), 15 min, Hettich Zentrifugen EBA 21).

The decanted solution and the precipitates were filled into separate Schlenk‐flasks which were connected to a Schlenk line. The flask containing the solution was subjected to the same processing steps as used previously described for the recrystallization of the electrolytes: heating to 120 °C for 2 h under vacuum (p ≈5–8⋅10^−2^ mbar), heating to 300 °C for 2 h under vacuum (p ≈5–8⋅10^−2^ mbar), and heating to 550 °C for 10 h in evacuated quartz ampoules (p ≈1 ⋅ 10^−3^ mbar). For the precipitates, heating to 120 °C under vacuum was sufficient to remove remaining EtOH.

### Characterization

#### X‐Ray Powder Diffraction and Rietveld Analysis

XRD patterns were recorded on a Rigaku SmartLab in Bragg‐Brentano geometry with Cu K_α_ radiation with a wavelength of 1.542 Å and a Hypix‐3000 detector. Samples were measured inside low background air‐tight sample holders (Rigaku), which were sealed inside an Ar‐filled glovebox.

Analysis of diffraction data was performed via the Rietveld method with the program TOPAS V.6.0. The instrumental intensity distribution of the diffractometer was determined empirically from a fundamental parameter set determined using a reference scan of LaB_6_ (NIST 660a). Microstructural parameters (i. e., crystallite size and strain broadening) were refined to adjust the peak shapes.

#### Scanning Electron Microcopy and Energy‐Dispersive X‐Ray Spectroscopy

Scanning electron microscopy (SEM) images were recorded using a secondary electron detector of a ZEISS EVO 15 microscope operating at 20 keV. Prior to the measurements, a layer of Au was sputter‐deposited onto the samples.

Additionally, energy‐dispersive X‐ray spectroscopy (EDX) measurements were conducted. The APEX software integrated with the SEM instrument was used for the analysis of the EDX data.

#### Electrochemical Impedance Spectroscopy

Electrochemical impedance spectroscopy (EIS) was performed using a TSC battery test cell (rhd instruments GmbH & Co. KG). Heating and cooling were achieved with a temperature‐controlled cell stand. For this, 50 mg of each powder was uniaxially pressed into a free‐standing pellet with a diameter of 7 mm (pelletizing pressure of 254 MPa for 5 min). The thickness of the obtained pellets was measured using calipers. Gold layers were sputter‐coated onto the surfaces. Measurements were performed using an electrochemical impedance analyzer NEISYS (Novocontrol Technologies) in a frequency range between 1 MHz and 0.1 Hz with a root‐mean‐square amplitude of 10 mV in a temperature range between 20 and 80 °C (ΔT=5 K, heating and cooling). Received data were analyzed using the software RelaxIS3 (rhd instruments GmbH & Co. KG).

#### Electrochemical Testing

For electrode preparation, 85 wt.% of pristine or recycled active electrode materials were mixed homogeneously with 5 wt.% Carbon Black Super P® (TIMCAL Ltd., Switzerland) as conducting additive and 10 wt.% polyvinylidene fluoride (Solef® PVDF, Solvay, Germany). The binder solution consisted of 10 wt.% PVDF in N‐methyl‐2‐pyrolidone (NMP, BASF, Germany). The obtained slurries were tape‐casted onto aluminum (for cathode materials) or copper (for anode material). After tape casting, the printed electrodes were dried at 55 °C for 24 h. Round electrodes with a diameter of 7.8 mm were cut out and the weight of the electrodes was measured. The electrodes were dried under vacuum in a Büchi oven (Büchi glass oven B‐585) at 80 °C for 24 h before being transferred without further contact with air into an argon‐filled glove box for cell assembly.

For testing of the electrode materials, cells of two‐electrode Swagelok®‐type set‐up were assembled. Reference/counter electrodes with a diameter of 8 mm were stamped out of Li foil (thickness 0.75 mm, Sigma Aldrich). Glass fiber membranes (Whatman GF/C) were used as separators, which were soaked in 180 μl electrolyte. As electrolyte a solution of 1 M LiPF_6_ in ethylene carbonate:dimethyl carbonate, ratio 1 : 1 (LP30, Merck KGaA, Germany) was used. The testing was performed with a VMP potentiostat (BioLogic Science Instruments). Cyclic voltammetry (CV) measurements were conducted in suitable potential ranges for the respective active electrode with a scan rate of 0.1 mVs^−1^_._

#### Inductively Coupled Plasma Mass Spectrometry

Quantitative elemental analysis of the electrode materials via inductively coupled plasma‐mass spectrometry (ICP‐MS) was conducted with an Agilent ICP‐MS system 7700. For sample preparation, ~8 mg of the respective material were dissolved in HCl and diluted using ultrapure water (Biochrom AG, sterile, suitable for HPLC). Argon 5.0 (Ar ≥99.999 mol %, ALPHAGAZ™ 1 Argon, Air Liquide) was used as plasma gas for ICP‐MS measurements. For calibration, a 28 multi‐element ICP‐MS standard solution (10 mg/l in 5 % HNO_3_, ROTI^®^Star) and Mulit‐Element Calibration Standard‐4 (10 mg/l in 5 %HNO_3_, Agilent Technologies) were used. An external calibration was done for quantification.

#### X‐Ray Photoelectron Spectroscopy

For the preparation of XPS samples, double‐sided adhesive tape, functioning in addition as an insulating layer, was used. Small quantity of powders of selected electrode materials were placed onto the tape which was previously stuck to the sample holder. Samples were transferred under Ar into the XPS chamber.

The X‐ray photoelectron data were recorded using a Kratos AXIS Ultra spectrometer and monochromatized Al Kα radiation. Survey spectra were acquired with a pass energy of 80 eV and detailed spectra with a pass energy of 20 eV. A charge neutralizer was used to compensate for the surface charging. The binding energies were calibrated to the C‐C peak of adventitious carbon in the C 1s spectrum at 284.8 eV.^[38]^ Data analysis was performed using the CasaXPS software.^[39]^

## Results and Discussions

Previous studies[^11^, ^12^, ^13^, ^14^, ^15^, ^18^] have reported that Li_6_PS_5_Cl, Li_6_PS_5_Br, and Li_6_PS_5_I can be synthesized via dissolution‐precipitation processes using various organic solvents. Additionally,, post‐processing of the pre‐synthesized SEs, involving complete dissolution (solution processing) is widely studied.[^17^, ^18^] EtOH is commonly employed as a solvent for both the synthesis and processing of argyrodite SEs.

This study focuses on the post‐processing of Li_6_PS_5_Cl, Li_6_PS_5_Br, and Li_6_PS_5_I to enable efficient separation of battery components, thereby exploring a potential recycling route. To examine the influence of electrolyte concentration and exposure duration to EtOH, a detailed investigation was conducted on Li_6_PS_5_Cl. Furthermore, the effect of solvent treatments with other organic solvents was representatively studied on Li_6_PS_5_Cl. This SE was selected as a representative material due to its stability and widespread use in solid‐state battery research, making it an ideal model for studying dissolution and interaction behaviors. By focusing on this SE, we were able to analyze a broader range of solvents and interactions in greater detail, providing a solid foundation for extending the methodology to other SE compositions. Finally, different electrode materials were introduced into the dissolution process to explore potential interactions between the dissolved argyrodite‐type electrolytes and the electrode materials.

### Influence of Dissolution and Recrystallization on Argyrodite‐Type Electrolytes

To qualitatively assess the solubilities of Li_6_PS_5_Cl, Li_6_PS_5_Br, and Li_6_PS_5_I in EtOH, different solid:liquid ratios were tested (Figure S 1). At relatively low concentrations (10 mg of electrolyte in 1 ml EtOH), the resulting clear solution containing Li_6_PS_5_Cl has a brown color, while the solutions with Li_6_PS_5_Br, and Li_6_PS_5_I display a yellow tint. These observations are consistent with previous findings.^[11]^ When the concentrations of the SEs are increased, complete dissolution is observed up to approximately 100 mg of electrolyte per 1 ml of EtOH. At higher concentrations, undissolved residuals remain present. For direct recycling of the SEs based on a dissolution‐based separation process, achieving complete dissolution of the SEs is crucial; therefore, higher concentrations of SEs should be avoided.

### Solvent Treatment of Li_6_PS_5_Cl

To study the impact of solvent treatment on Li_6_PS_5_Cl, a range of organic solvents with different physical and chemical properties, covering a spectrum from non‐polar over polar aprotic to polar protic solvents were used. The physical and chemical properties of the solvents, along with the solubility status of the SE in each solvent, are provided in Table S 1. Given that the polar protic alcohol EtOH is most commonly employed for the solution processing of Li_6_PS_5_Cl, a focus was set on this solvent.

**Figure 2:**
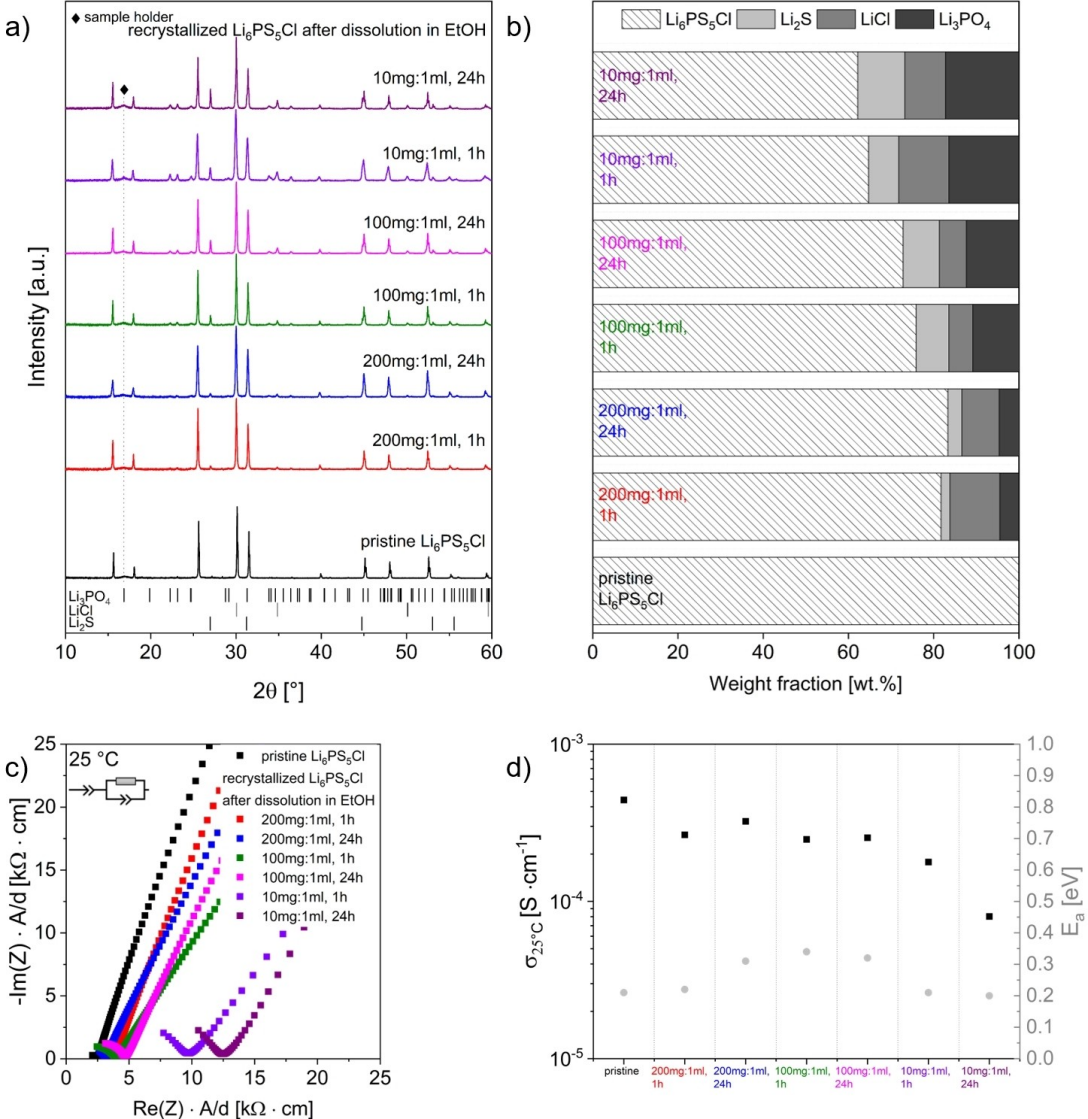
a) X‐ray diffraction patterns of recrystallized samples in comparison to pristine Li_6_PS_5_Cl after dissolution using different solid:liquid ratios of Li_6_PS_5_Cl to EtOH and reaction times. The samples were heated to 550 °C for 10 h in evacuated quartz ampoules. The positions of the characteristic reflection ticks of decomposition products Li_2_S, LiCl, and Li_3_PO_4_ observed after the dissolution and recrystallization are given; b) Composition of recrystallized samples in comparison to pristine Li_6_PS_5_Cl after the dissolution and recrystallization.; c) Nyquist plots; d) Ionic conductivities σ_25 °C_ and activation energies E_A_.

An X‐ray diffraction study on Li_6_PS_5_Cl after dissolution using different solid:liquid ratios of 200 mg:1 ml, 100 mg:1 ml, and 10 mg:1 ml of SE to EtOH with processing times of 1 and 24 h (Figure 2 a) was conducted. The results reveal that the main phase formed after recrystallization at 550 °C is Li_6_PS_5_Cl. However, additional phases such as Li_2_S, LiCl, and Li_3_PO_4_ are also present. Quantitative phase analysis and cell parameter determination were obtained from Rietveld analysis (Figure 2 b, Figure S 2 for exemplary Rietveld refinements, Table S 2 for quantitative analysis and refined cell parameters). No significant differences in the lattice parameters between pristine and recrystallized Li_6_PS_5_Cl were observed, suggesting minimal structural changes after the dissolution‐precipitation process.

A dependence of the weight fractions of the obtained phases on the concentrations and exposure times is observed. Lower concentrations of Li_6_PS_5_Cl in EtOH and longer stirring times result in higher phase fractions of undesired decomposition products; however, the concentration plays a more significant role compared to exposure time in determining the final phase composition. Notably, the progressive increase inLi_3_PO_4_ formation when lowering the concentration suggests that nucleophilic reactions between EtOH and the PS_4_^3−^ anions in Li_6_PS_5_Cl increase. Lower concentrations correspond to reduced amounts of PS_4_^3−^ anions and a relatively higher proportion of OH groups (see also Table S 3 for ratios of PS_4_^3−^ anions to OH groups across the different concentrations studied).The decomposition mechanism of PS_4_^3−^ anions has been detailed by Hatz et al.^[40]^: the nucleophilic attack of EtOH on PS_4_^3−^ leads to stepwise substitution of sulfur with oxygen. This degradation has been also reported for the synthesis of argyrodites in EtOH.^[12]^ With the formation of oxygen‐substituted thiophosphate Li_3_PS_4–x_O_x_, phase fractions of Li_2_S and LiCl also emerge, resulting from the decomposition of Li_6_PS_5_Cl into these three phases. A comparison of the cell volumes of Li_3_PO_4_ (~316.3 Å^3^)^[41]^ and Li_3_PS_4_ (~638.92 Å^3^) suggests that the formed Li_3_PS_4–x_O_x_ (ranging from ~316.2 Å^3^ to ~317.6 Å^3^, depending on concentration, as shown in Table S 2) is oxygen‐rich. This suggests that concentration plays a critical role in optimizing process parameters to minimize decomposition. Furthermore, the use of an alternative solvent may help to reduce decomposition caused by nucleophilic attack (as discussed further below).

The observed compositional changes are expected to have an influence on the ionic conductivity of the recrystallized SE materials. Therefore, electrochemical impedance spectroscopy measurements were performed to assess these changes. The Nyquist plots of pristine, and recrystallized Li_6_PS_5_Cl after dissolution and recrystallization (measured at 25 °C) are shown in Figure 2 c (representative data fits and fitted parameters are provided in Figure S 3 a and b). A fitting model consisting of one parallel resistor (R) and one constant phase element (CPE) in series with an additional CPE was used for fitting the Li‐ion transport process and the electrode‐ion‐blocking effect at the electrode. Since the contributions of the bulk and the grain boundaries cannot be deconvoluted in the investigated temperature range, the (R/CPE) element is assigned to the total resistance of the SE.^[42]^ The room temperature ionic conductivity values and activation energies determined from temperature‐dependent measurements are shown in Figure 2 d; the Arrhenius plots are provided in Figure S 3 c. In agreement with previous studies,[^43^, ^44^] the treatment of Li_6_PS_5_Cl with EtOH leads to an increase in resistance. A progressive decrease in ionic conductivities with lower concentrations of Li_6_PS_5_Cl (and longer exposure times) to EtOH is observed which is also in agreement with the increase of phase fractions of decomposition phases. The ionic conductivities for the higher and middle concentrations remain in the same order of magnitude, though they decrease to approximately half of the values observed for the pristine electrolyte. Comparatively, the strongest decrease is found for the recrystallized sample that underwent dissolution in EtOH with a solid:liquid ratio of 10 mg:1 ml and a stirring time of 24 h. Interestingly, independently on the dissolution conditions applied, the pristine and all recrystallized samples show capacitances in the order of 10^−11^ F. However, the lower α‐values of recrystallized Li_6_PS_5_Cl indicate a more complex underlying process compared to pristine Li_6_PS_5_Cl, suggesting significant contribution of grain boundary resistance to the total conductivity.^[45]^ Furthermore, electrolyte‐electrode interface contributions (10^−7^–10^−5^ F) can be identified.^[46]^ Differences between the samples are likely related to a subtle change in the microstructure (see SEM investigation below), as the particle size and presence of agglomerates after recrystallization can change the mechanical properties and, thus, interface properties at the electrodes. The activation energies of the recrystallized SE materials partially increase compared to the Li_6_PS_5_Cl, with variations of up to approximately 150 meV. Providing a comprehensive explanation on the observed changes in conductivity and activation energy is challenging due to the need to consider a wide range of effects. They are likely the result of a complex interplay between different factors, such as interactions between impurity/decomposition phases and the desired argyrodite phase. Variations in crystallinity, influenced by the extent to which impurity/decomposition phases affect the recrystallization of the argyrodite SE, are likely, and depend strongly on the quantities and the distribution of these phases. Additionally, factors such as tortuosity may also contribute to the observed differences, particularly when considering the increased presence of grain boundaries and phase interfaces, which can further impede charge transport. A more detailed understanding would require further studies, such as the development of model mixtures with varying phase compositions and crystallinities, which is beyond the scope of this study.

**Figure 3:**
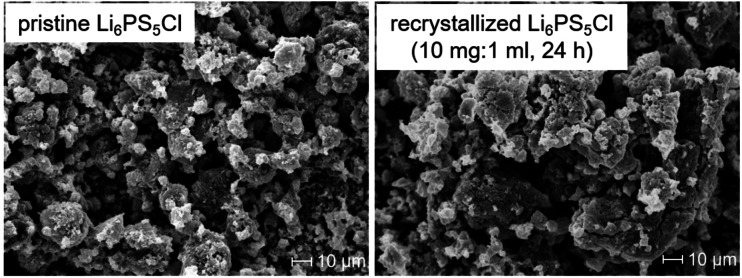
SE micrographs of pristine and recrystallized Li_6_PS_5_Cl after the dissolution in EtOH (solid:liquid ratio of 10 mg:1 ml, stirring time of 24 h). The recrystallized sample was heated to 550 °C for 10 h in an evacuated quartz ampoule.

Morphological changes of Li_6_PS_5_Cl after the dissolution and recrystallization in comparison to pristine Li_6_PS_5_Cl were assessed via scanning electron microscopy (Figure 3). Minor differences in morphologies are observed with both SE powders consisting of highly agglomerated particles with a relatively wide particle size distribution. A higher fraction of larger agglomerates is found for recrystallized Li_6_PS_5_Cl.

**Figure 4:**
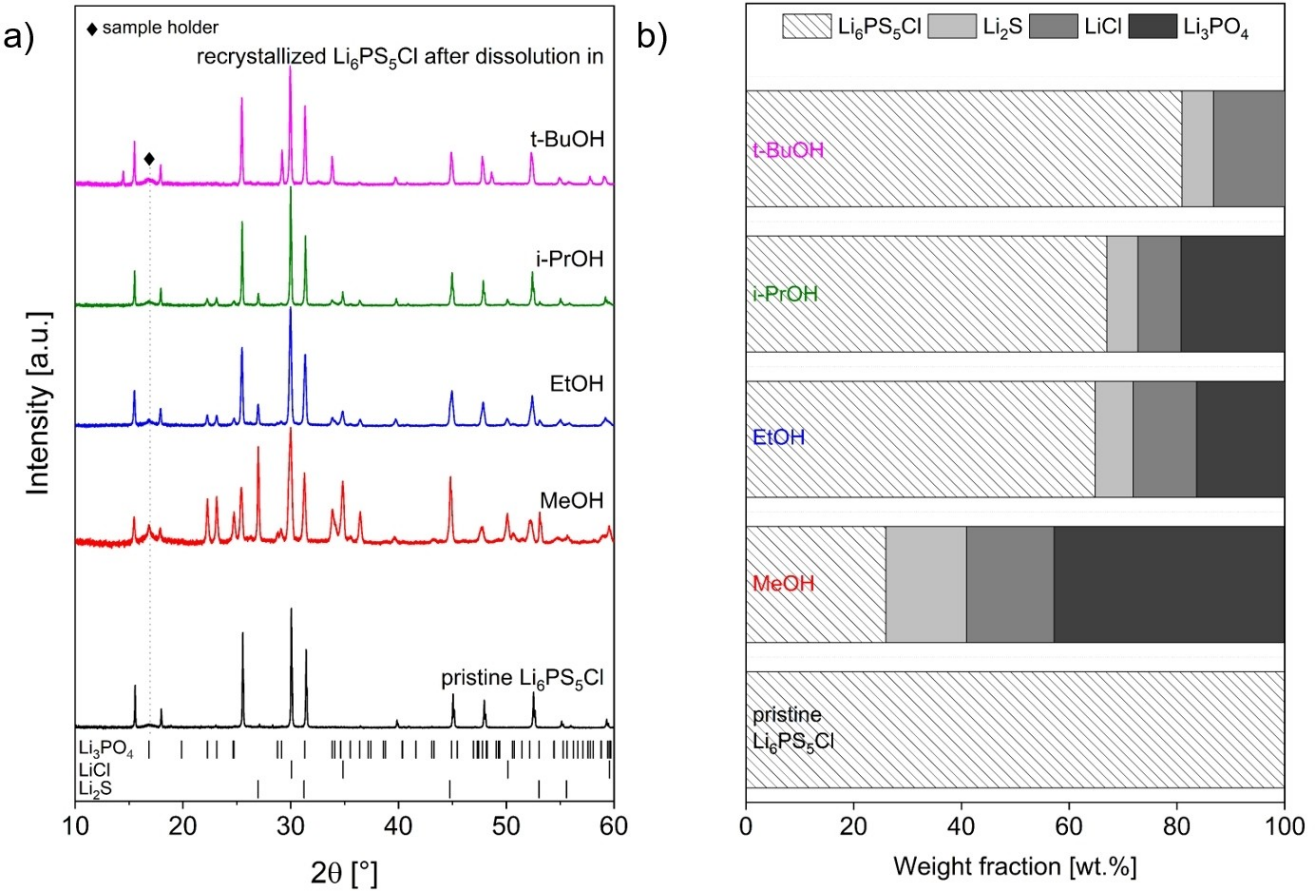
a) X‐ray diffraction patterns of recrystallized samples in comparison to pristine Li_6_PS_5_Cl after dissolution in MeOH, EtOH, i‐PrOH, and tert‐BuOH (solid:liquid ratio of 10 mg:1 ml, stirring time of 24 h). The samples were heated to 550 °C for 10 h in evacuated quartz ampoules. The positions of the characteristic reflection ticks of decomposition products Li_2_S, LiCl, and Li_3_PO_4_ observed after the dissolution and recrystallization are given; b) Composition of recrystallized samples in comparison to pristine Li_6_PS_5_Cl after the solvent treatment.

As has been previously reported,^[16]^ obtaining crystalline Li_6_PS_5_Cl after dissolution in EtOH requires a heat treatment at relatively high temperature (T >500 °C). Without this heat treatment, complete decomposition of the argyrodite crystal structure under the formation of phase mixtures of Li_2_S, LiCl, and other (partially amorphous) phases is observed. Similar findings were made when using alcohols other than EtOH (for example MeOH).^[43]^ To investigate the influence of MeOH, i‐PrOH, and t‐BuOH on the dissolution and recrystallization behavior of Li_6_PS_5_Cl, a further series of experiments was conducted (Figure 4). The used alcohols differ in their chain length and in the arrangement of the various groups relative to each other (primary, secondary, and tertiary alcohols were considered), which result in different effects due to steric hindrance depending on the size of the molecule.^[40]^ Thus, the dissolution of Li_6_PS_5_Cl is hindered since the nucleophilic OH group of the alcohol cannot approach the PS_4_^3−^ anions sufficiently and the solvation of the anions is not possible. This might also reduce the overall decomposition of the SE via nucleophilic attack. The conducted dissolution experiments confirm this influence: While Li_6_PS_5_Cl dissolves completely in MeOH and EtOH, small amounts of undissolved residuals are observed in i‐PrOH; the electrolyte does not dissolve at all in t‐BuOH. X‐ray diffraction measurements after the removal of t‐BuOH (without additional heating, not shown) confirms that only for this solvent the Li_6_PS_5_Cl structure remains intact. For the other alcohols, significant decomposition is observed. To recrystallize Li_6_PS_5_Cl, an additional heating step at 550 °C is required. However, considerable amounts of Li_3_PO_4_ (and corresponding weight fractions of Li_2_S and LiCl) are formed as an unwanted impurity phase. The phase fractions and lattice parameters of the formed phases after the thermal treatment are reported in Table S 4. In accordance with the molecule size of the alcohol, the highest degree of decomposition and the largest phase fraction of Li_3_PO_4_ is found in SE recrystallized from the MeOH solution. Additionally, similar to the findings made with varying concentrations of Li_6_PS_5_Cl in EtOH, higher amounts of OH groups in relation to PS_4_^3−^ anions when using the different alcohols also influences the degradation of Li_6_PS_5_Cl (Table S 3).

It should be mentioned that dissolution of Li_6_PS_5_Cl can be also achieved using a mixture of an alcohol (again most often EtOH is used) and another organic solvent.[^13^, ^47^, ^48^, ^49^] However, without the addition of an alcohol, the formation of suspensions with little to no dissolution of the SE is observed. For example, the use of a pure non‐polar solvent such as hexane or of polar aprotic solvents (e. g., THF, EA, or ACN) results in the formation of suspensions with little to no dissolution of the SE. In accordance with Ruhl et al.,^[43]^ for treatments in these solvents, the crystal structure remains relatively unchanged due to absence of dissolution, and crystalline Li_6_PS_5_Cl is recovered after the removal of the solvent at comparatively low temperatures (no heat treatment at T >500 °C is required). The XRD patterns are shown in Figure S 4 a. Minor decomposition under the formation of Li_2_S, LiCl, and amorphization takes place. Interestingly, besides the aforementioned alcohols, NMF was found to fully dissolve Li_6_PS_5_Cl. Compared to more nucleophilic alcohols with OH bonds, using this strongly polar, weakly protic solvent containing NH bonds instead could be beneficial in reducing undesired decomposition that leads to formation of PS_4‐x_O_x_ units. The weaker proton‐donating ability of NH bonds, combined with their lower nucleophilicity, helps minimize side reactions, stabilizing the desired reaction intermediates and preventing the formation of unwanted byproducts. In agreement with this, lower phase fractions of Li_3_PO_4_ (~2.5 wt.%) are found after the removal of NMF and recrystallization compared to the dissolution treatments in alcohols. As previously reported for β‐Li_3_PS_4_ dissolved in NMF_,_ ^[50]^ the formation of an intermediate NMF complex phase is observed before recrystallization at higher temperatures. The similarity of the diffraction patterns (Figure S 4 b) suggests a close structural relation of this complex with Li_3_PS_4_ ⋅ 2NMF, which is a layered compound with layers of composition Li_3_PS_4_⋅2NMF.^[50]^ Consistent with this and the overall composition of Li_6_PS_5_Cl, LiCl and additional reflections of an unidentified phase (possibly related to Li_2_S) are found besides this complex phase. The decomposition of this complex, leading to a (partial) reformation of Li_6_PS_5_Cl can be achieved via a heat treatment at temperatures as low as ~300 °C under vacuum. This is significantly lower compared to the recrystallization step at 500 to 550 °C needed after the dissolution in alcohols. After heating, ~66 wt.% of Li_6_PS_5_Cl is obtained, which is comparable to the phase fraction of Li_6_PS_5_Cl obtained after dissolution in EtOH However, the significantly reduced amount of otherwise irreversibly formed Li_3_PO_4_, due to fewer nucleophilic attacks, along with the lower temperatures required for recrystallization, underscores the potential of NMF as a solvent for dissolution‐based recycling approaches. To reduce the phase fractions of Li_2_S (~19 wt.%), LiCl (~12.5 wt.%), and Li_3_PO_4_ (~2.5 wt.%) as well as of potentially existing amorphous phases, further optimization of the heating procedure would be required.

### Solvent Treatment of Li_6_PS_5_Br and Li_6_PS_5_I

**Figure 5:**
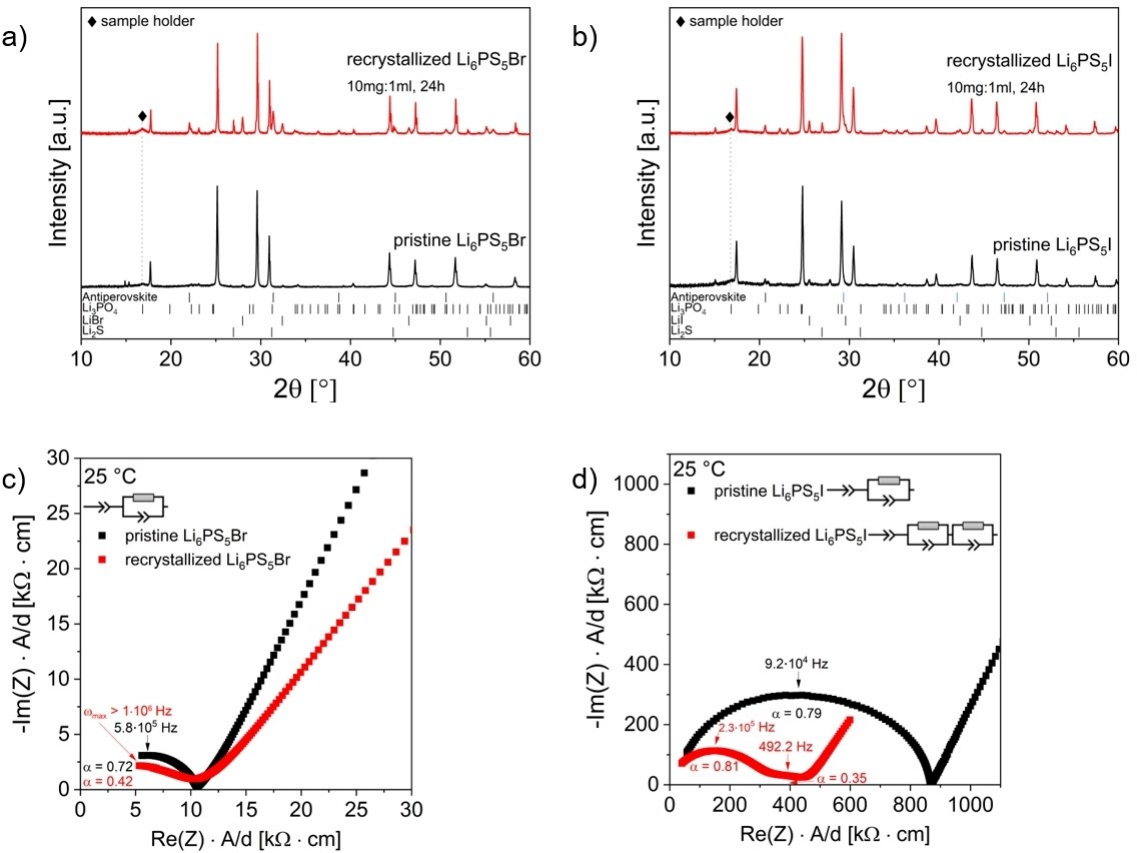
X‐ray diffraction patterns of recrystallized sample in comparison to pristine Li_6_PS_5_Br (a) and Li_6_PS_5_I (b) after dissolution in EtOH (solid:liquid ratio of 10 mg:1 ml, stirring time of 24 h). The samples were heated to 550 °C for 10 h in evacuated quartz ampoules. The positions of the characteristic reflection ticks of decomposition products Li_2_S, LiBr, LiI, Li_3_PO_4_, antiperovskite phases Li_3_OBr/Li_2_(OH)Br/LiBr(H_2_O) and Li_3_OI/Li_2_(OH)I/LiI(H_2_O) observed after the dissolution and recrystallization are given. Nyquist plots with corresponding fit model of pristine and recrystallized Li_6_PS_5_Br (c) and Li_6_PS_5_I (d).

The effects of solvent treatments of Li_6_PS_5_Br and Li_6_PS_5_I using EtOH were investigated using a solid:liquid ratio of 10 mg:1 ml of SE to EtOH with a reaction time of 24 h. This choice of reaction conditions was based on the fact that for this concentration complete dissolution (without excessive reagent use) is assured, while also maximizing potential interactions between the SE and the solvent, which were a primary focus of this study. The X‐ray diffraction patterns of the pristine and recrystallized SEs (Figure 5 a and b) reveal that the main phases formed after dissolution and heat treatment correspond to Li_6_PS_5_Br and Li_6_PS_5_I. Next to the expected argyrodite‐type phases, Li_2_S, LiBr or LiI, and Li_3_PO_4_ are present, indicating again a partial recrystallization. However, in contrast to Li_6_PS_5_Cl, next to these phases, the formation of additional phases with antiperovskite structure is observed for both SEs. These phases could be Li_3_OBr, Li_2_Br(OH) or LiBr(H_2_O) and Li_3_OI, Li_2_I(OH), or LiI(H_2_O), respectively.[^51^, ^52^, ^53^] An unambiguous identification of the composition of these phases based on XRD measurements only is not possible due to the similar (and comparatively low) scattering factors of Li and H. The phase fractions and lattice parameters of the formed phases are given in Table S 6 and S 7. Overall similar phase fractions of the argyrodite‐type phases (~65 wt.%) and decomposition products (~35 wt.%) are found for all three SEs when using the same solid:liquid ratios and reaction time. Morphological changes due to the dissolution and recrystallization process were investigated by SEM (Figure S 5). As for pristine and recrystallized Li_6_PS_5_Cl, highly agglomerated particles with largely differing sizes are found. For pristine and recrystallized Li_6_PS_5_Br, no significant differences are observed. However, recrystallized Li_6_PS_5_I possesses significantly smaller particles compared to pristine Li_6_PS_5_I, indicating an impact of the synthesis approach and material processing (mechanochemical vs. a dissolution‐based synthesis) for this compound.

The solvent treatment is found to partially alter the impedance response (Figure 5 c and d). Interestingly, for Li_6_PS_5_I, a higher ionic conductivity is observed after post‐treatment. This indicates that the dissolution and subsequent recrystallization process can enhance the intrinsic properties of the SE, even though impurity phases with lower ionic conductivities are present in addition. This could be due to the reorganization of the crystal structure or removal of amorphous regions. Moreover, the observed change in morphology might have a beneficial influence.

For pristine and recrystallized Li_6_PS_5_Br, the same fitting model used for Li_6_PS_5_Cl–comprising one parallel R and CPE in series with an additional CPE–can be used; the fits and the Arrhenius plots are shown in Figure S 6. At 25 °C, ionic conductivities of 1.68 ⋅ 10^−4^ S ⋅ cm^−1^ and 1.59 ⋅ 10^−4^ S ⋅ cm^−1^ are observed for pristine and recrystallized Li_6_PS_5_Br, respectively. The activation energy is 0.29 eV for both samples. The corresponding capacitances remain in the same order of magnitude (10^−11^ F) for pristine and recrystallized Li_6_PS_5_Br, suggesting similar behavior, though the α‐values differ significantly.

For Li_6_PS_5_I, in agreement with its crystal structure, overall lower ionic conductivities and higher activation energies are found (2.00 ⋅ 10^−5^ S ⋅ cm^−1^ and 0.35 eV for pristine Li_6_PS_5_I and 3.20 ⋅ 10^−5^ S ⋅ cm^−1^ and 0.43 eV for recrystallized Li_6_PS_5_I). Interestingly, the capacitance of 10^−9^ F of the pristine material suggest a large contribution of the grain boundaries to the total resistance. In contrast to this, for recrystallized Li_6_PS_5_I, the fitting model had to be modified to two parallel R and CPE in series with an additional CPE, and distinct bulk (10^−11^ F) and grain boundary (10^−9^) contributions can be identified.^[46]^

### Investigation of Degradation of Argyrodite Electrolytes and Electrode Materials

#### Degradation of Argyrodite Electrolytes due to Electrode Materials in Dissolution‐Based Separation Process

The presence of electrode materials (investigated electrode materials are LCO, LMO, NMC811, LFP, and LTO) during the dissolution process may increase the risk for side reactions.^[28]^ To investigate the influence of individual electrode materials on the recrystallization behavior of the Li_6_PS_5_Cl, Li_6_PS_5_Br, and Li_6_PS_5_I, both fractions were mixed, and the SE was dissolved in EtOH. The unsolvable electrode materials were then separated using centrifugation. Recrystallization of the separated SE was achieved using the same heating parameters as utilized before. The influence on the electrode materials is discussed separately in Section 3.2.2.

**Figure 6:**
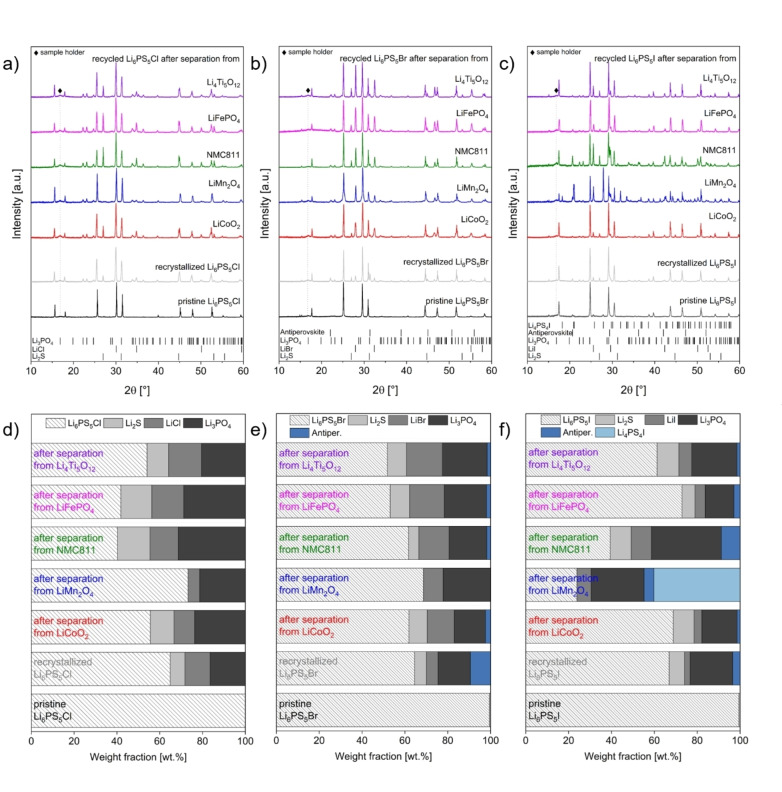
X‐ray diffraction patterns of recycled samples in comparison to pristine and recrystallized Li_6_PS_5_Cl (a), Li_6_PS_5_Br (b) and Li_6_PS_5_I (c) after dissolution in EtOH (solid:liquid ratio of 10 mg:1 ml, stirring time of 24 h) and separation from different electrode materials. The samples were heated to 550 °C for 10 h in evacuated quartz ampoules. The positions of the characteristic reflection ticks of decomposition products Li_2_S, LiBr, LiI, Li_3_PO_4_, the anitperovskite phases Li_3_OBr/Li_2_Br(OH)/LiBr(H_2_O) and Li_3_OI/Li_2_BI(OH)/LiI(H_2_O), and Li_4_PS_4_I observed after the dissolution and recrystallization are given. Composition of recycled samples in comparison to pristine and recrystallized Li_6_PS_5_Cl (d), Li_6_PS_5_Br (e) and Li_6_PS_5_I (f) after the dissolution in EtOH (solid:liquid ratio of 10 mg:1 ml, stirring time of 24 h) and separation from different electrode materials.

To gain insights into potential interactions between the dissolved SEs and the electrode materials, an X‐ray diffraction study was conducted. By comparing the diffraction patterns of the recycled electrolytes after recrystallization to those of the pristine and recrystallized materials, significant differences are observed depending on the electrode material used (Figure 6a–c). Partially considerable increases in the phase fractions of the decomposition products Li_2_S, LiCl/LiBr/LiI, Li_3_PO_4,_ and the antiperovskite phases are observed. In addition, an influence related to the argyrodite electrolyte used is found which is reflected in variations in the relative amounts of the different phases and formation of additional phases. This is related to the presence of the different halide ions in solution. Results of the quantitative phase analysis determined using Rietveld analysis are illustrated in Figure 6 for the different SEs (see also Tables S 5‐S 7). Based on X‐ray diffraction, only crystalline phases can be identified. However, it should be mentioned that the ongoing interactions also lead to significant amorphization. Furthermore, it is important to recognize that during dissolution, elemental transfer from the electrode materials to the SEs is likely, and this process could significantly influence the ionic conductivity of the SEs. In our previous study on the dissolution‐based separation of β‐Li_3_PS_4_ from the same electrode materials, we demonstrated that increased concentrations of Mn, Fe, Co and Ni were present in the recycled electrolyte.^[28]^ A similar elemental transfer can be anticipated for the materials examined in this study. This is supported by initial visual evidence: when dissolving the SEs separated from LMO and LFP in water, the solution exhibits brown and orange‐brown discoloration, respectively, characteristic for the presence of Mn and Fe ions.^[54]^

Several trends can be identified based on X‐ray diffraction, which have a complex dependence on competing interactions occurring during the dissolution. Compared to the recrystallized materials (and to other electrode materials), a relatively small increase in decomposition products is found for recycled Li_6_PS_5_Cl and Li_6_PS_5_Br after the separation from LCO and LTO. This indicates that the SEs can be recovered after the dissolution‐based separation process in a similar quality (at least with respect to the formed crystalline phases) compared to the recrystallized materials. Recycled Li_6_PS_5_I separated from LCO contains even slightly reduced fractions of these products.

For the SEs separated from LMO, NMC811 and LFP, more complex interactions with strong differences between the used electrode material as well as the argyrodite electrolytes are found. For LMO, a distinctive degradation mechanism can be deduced: After its separation from Li_6_PS_5_Cl and Li_6_PS_5_Br, no Li_2_S and no lithium‐containing antiperovskite phase are found. This indicates a topochemical reaction between LMO and the ions present in these phases (most probably of Li ions, see also Section 3.2.2 for chemical lithiation of LiMn_2_O_4_), leading to their consumption during the dissolution process, which is in agreement with the highest reductivity of Li_2_S amongst the decomposition products. For recycled Li_6_PS_5_I, this effect is even more pronounced, leading to an even higher fraction of side products: while this sample also contains no additional Li_2_S, the formation of ~40 wt.% Li_4_PS_4_I (contains one molar equivalent less Li_2_S than Li_6_PS_5_I) next to only ~24 wt.% Li_6_PS_5_I is found. Even though I^−^ is a weaker reductant than S^2−^, it likely promotes the reduction kinetics (compare for example its influence in redox titrations^[55]^). This might facilitate the reduction of LiMn_2_O_4_ under the formation of I_2_. However, according to their standard potentials, I_2_ is not stabile against S^2−^. Thus, the re‐formation of I^−^ might occur, which leads to the formation of iodide‐containing phases such as Li_4_PS_4_I and anitperovskite phases, which do not contain free S^2−^ species.

For NMC811 and LFP, other factors influence the phase composition after the dissolution. It should be noted that topochemical reactions as observed for LMO are not possible for these compounds. Instead, Ni‐rich cathodes such as NMC811 show instability due to the preferred reduction of Ni^3+^ to Ni^2+^ (compare also degradation in ambient air)[^56^, ^57^]. This is in particular pronounced in the presence of S^2−^ as well as I^−^, since Ni^3+^ is not stable towards these species. These redox reactions lead to a considerable increase in the phase fractions of the decomposition products found in recycled Li_6_PS_5_Cl and Li_6_PS_5_I. In contrast, LFP contains only Fe^2+^. Thus, it should not undergo any redox reactions with the electrolyte constituents. Remarkably, the recycled Li_6_PS_5_I can be recovered with lower amounts of decomposition products compared to the recrystallized material. For recycled Li_6_PS_5_Cl and Li_6_PS_5_Br, on the other hand, considerably higher phase fractions of the decomposition products are found. The presence of relatively large amounts of Li_2_S and LiCl/LiBr might indicate that different stabilities and solubilities of the potentially formed complexes have a significant influence. In general, I^−^ does not tend to build complexes with transition metal species in solution, which might limit acid‐base type reactions of Li_6_PS_5_I with the different electrode materials in the absence of redox interactions.^[58]^

These compositional changes can have significant influence on the functional properties of the recycled electrolyte materials. To examine the effects of SEs degradation on the ionic conductivities, electrochemical impedance spectroscopy was exemplarily performed on the recycled Li_6_PS_5_Cl samples separated from the various electrode materials (Figure S 7). The observed ionic conductivities are in general agreement with the observed changes in composition. In the case of recycled Li_6_PS_5_Cl separated from LCO, LMO and LTO, larger phase fractions of Li_6_PS_5_Cl can be related to higher ionic conductivities. Compared to pristine and recrystallized Li_6_PS_5_Cl, the capacitances are with 10^−10^ F one order of magnitude larger (see Section 3.1.1), indicating a stronger influence of grain boundary resistances. The SE separated from NMC811 and LFP show significant lower conductivities. The activation energies of the separated SE samples differ significantly from those of pristine and recrystallized Li_6_PS_5_Cl, covering a range between 0.28 to 0.44 eV. While the precise origin of these changes cannot be determined due to the complexity of the effects, they are likely influenced by the inclusion of trace elements from the electrodes, which may affect crystallization behavior, grain boundary resistances, and tortuosity.

#### Degradation of Electrode Materials Due to Argyrodite Electrolytes in Dissolution‐Based Separation Process

**Figure 7:**
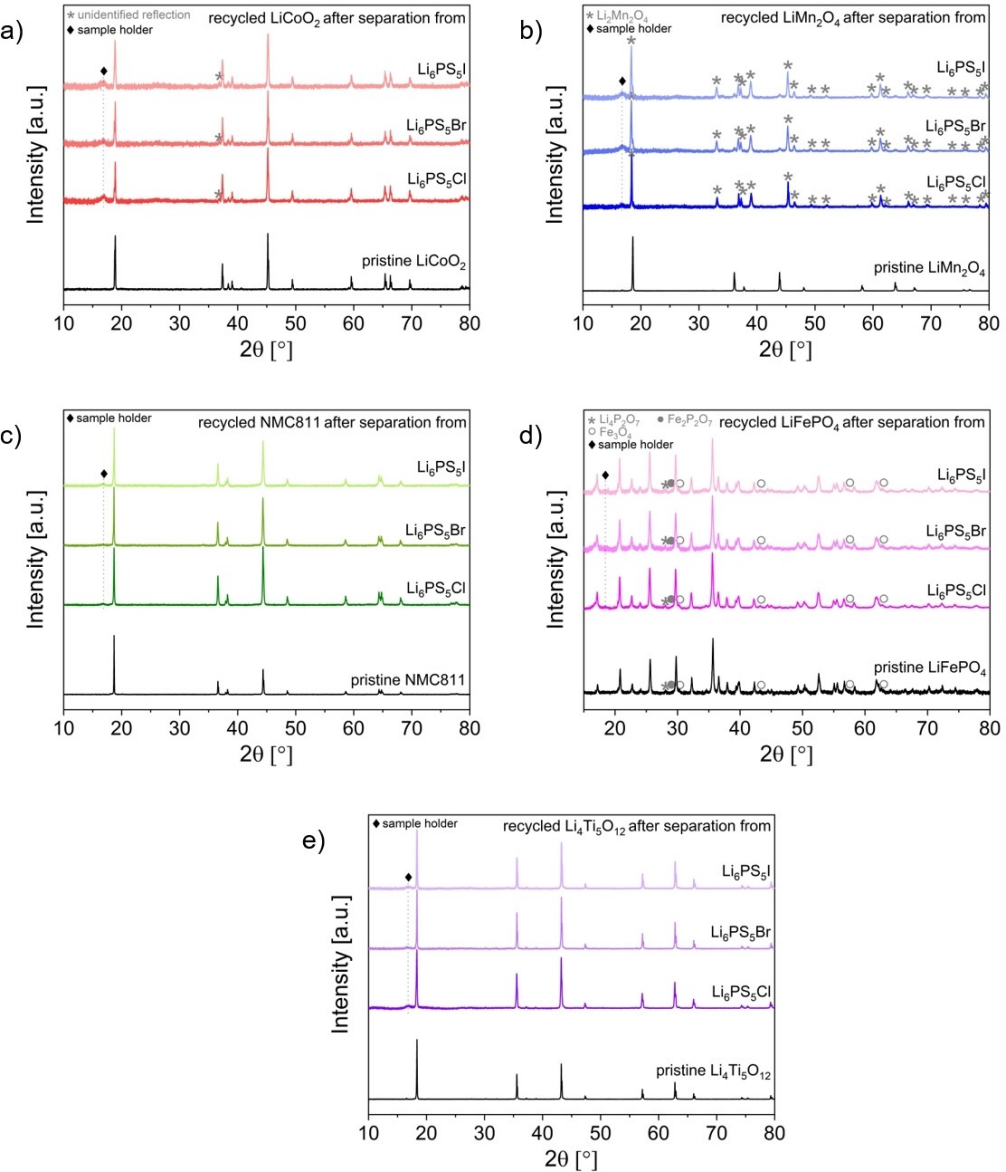
X‐ray diffraction patterns of recycled electrode materials after separation from Li_6_PS_5_Cl, Li_6_PS_5_Br, and Li_6_PS_5_I in comparison to pristine LiCoO_2_ (a), LiMn_2_O_4_ (b), NMC811 (c), LiFePO_4_ (d) and Li_4_Ti_5_O_12_ (e). For the dissolution of the SEs, EtOH was used (solid:liquid ratio of 10 mg:1 ml, stirring time of 24 h).

The different observed degradation interactions between the dissolved SEs and electrode materials are likely to be reflected in changes of the composition of the recycled electrode materials. Like the recycled SEs, these changes can significantly impact the electrochemical properties of the recycled electrode materials. This was initially assessed via X‐ray diffraction (Figure 7, Figure S 8 for exemplary Rietveld refinements, Table S 8 for quantitative analysis and refined cell parameters). The XRD patterns of LCO (except for one unidentified reflection at ~36.8° 2θ), NMC811, LFP (phase fractions of impurity phase remain constant in pristine and recycled LFP) and LTO remain relatively unchanged. However, significant alterations are observed between the patterns of pristine and recycled LMO. These findings are in general agreement with our previous study on the separation of β‐Li_3_PS_4_ from these electrode materials.^[28]^ Moreover, the presence of additional Li_2_S and LiCl/LiBr/LiI does not lead to any differences in the bulk composition of a specific material. Notably, no additional crystalline phases, such as transition metal sulfides or halides, can be identified in any of the XRD measurements. It is important to note that quantification errors in Rietveld analysis are around 1–2 wt %. Therefore, the phase fractions of these phases might be too low to be detected (e. g., formation of surface layers of the electrode particles). For more precise quantification, ICP‐MS measurements were performed; investigated elements included Li, P, Mn, Fe, Co and Ni. Based on this, the overall stoichiometry of the electrode materials could be confirmed (Table S 9). For all recycled electrode materials (except for NMC), an increase of the Li content is observed. Strongest deviations of the Li content are found for recycled LMO, for which large fractions of the active material are transformed into Li_1+x_Mn_2_O_4_ after the separation from all three SEs. As has been reported before, this phase can be prepared by various chimie douce techniques, including chemical lithiation using for example n‐butyl lithium[^59^, ^60^] or LiI^[61]^ as reducing agents. Thus, during the dissolution of the argyrodite electrolytes, Li ions intercalate into the spinel structure of LiMn_2_O_4_, which is associated with a reduction in the Mn oxidation state from +III/IV in LiMn_2_O_4_ to +III in Li_2_Mn_2_O_4_. This transformation can be also confirmed by the color change of the recycled LMO from black, characteristic for LiMn_2_O_4_, to brown (Figure S 9).^[60]^ However, this lithiation leads to a Li deficiency in the electrolytes after separation and recrystallization, implying also a considerable amorphization, though the ionic conductivity remains comparatively high (see Section 3.2.1). Thus, the interactions between the dissolved SEs and LMO substantially affect both the SE and the electrode material. In contrast, the other electrode materials exhibit less significant increases in the Li content. Rather than Li ions being intercalated (no structural changes are observed for these phases after the separation), it is more likely that Li appears in impurity or decomposition phases, such as Li_2_S, on the particle surfaces. In addition, a cross transfer of phosphorous species from the SEs to the electrodes was observed (compare EDX and XPS results of recycled NMC811 and LFP discussed below; these measurements also indicate the transfer of S, Cl, Br and I ions).

**Figure 8:**
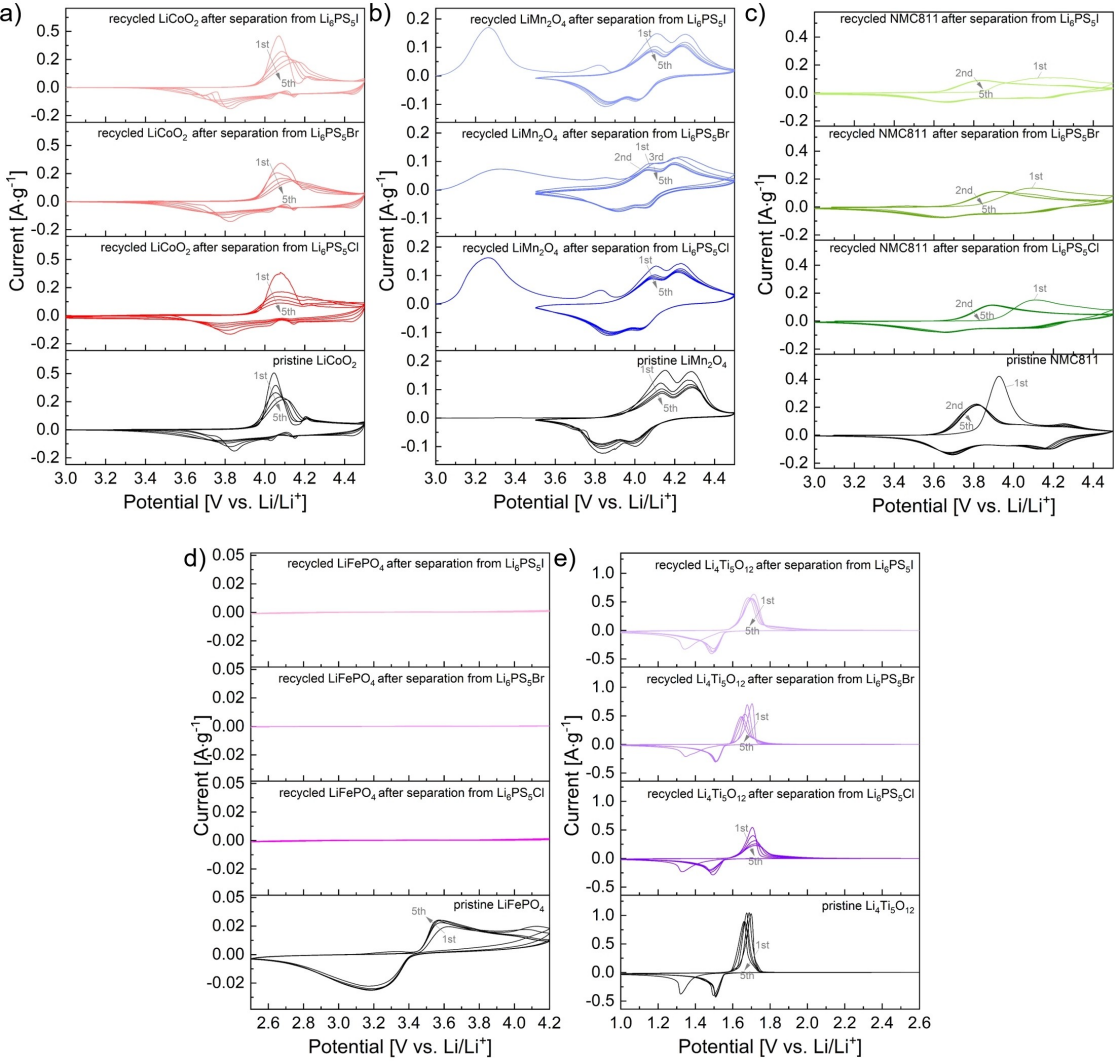
Cyclic voltammograms of recycled electrode materials after separation from Li_6_PS_5_Cl, Li_6_PS_5_Br, and Li_6_PS_5_I in comparison to pristine LiCoO_2_ (a), LiMn_2_O_4_ (b), NMC811 (c), LiFePO_4_ (d), and Li_4_Ti_5_O_12_ (e). For the dissolution of the SEs, EtOH was used (solid:liquid ratio of 10 mg:1 ml, stirring time of 24 h).

Changes of the electrochemical behavior of the recycled materials, attributed to the dissolution‐based separation process, were assessed using cyclic voltammetry (CV) measurements and compared to pristine materials (Figure 8). For this, Li‐ion batteries with an organic liquid electrolyte and tape‐casted electrodes were assembled and cycled against metallic lithium. In agreement with the observed relatively small morphological and compositional changes of both the recycled electrodes and electrolytes, comparatively good cycling behavior is found for recycled LCO and LTO, even though broader oxidation and reduction peaks with overall lower current maxima/minima are observed compared to the pristine materials. The oxidation and peak potentials of pristine and recycled LCO and LTO are listed Tables S 10 and 11. The broader peaks may be due to the presence of multiple species that oxidize and reduce at similar potentials. While no significant changes in the crystal structure and composition of these electrode materials are found after the separation from the argyrodite SEs, this suggests that the dissolution process does still affect the electrode materials, potentially due to interfacial reactions on the particle surfaces.

Stronger deviations in the oxidation and reduction curves are found for recycled LMO, NMC811, and LFP. For LMO and NMC811, the main differences between the pristine and recycled materials are found during the first oxidation with additional oxidative signals (compare Tables S 12‐S 14 for oxidation and reduction peak potentials). The following cycles are fairly similar; however, the oxidation and reduction peaks are partially shifted and broader compared to the pristine materials. In addition, the peak currents are again lower. The additional oxidation peaks observed during the first charging of recycled LMO are related to the delithiation of Li_1+x_Mn_2_O_4_, which is formed during the dissolution process, to LiMn_2_O_4_. Limiting the discharge potential to ≥3.5 V prevents the formation of fully lithiated Li_2_Mn_2_O_4_ during subsequent discharge steps. As a result, only partially discharged LiMn_2_O_4_ is formed and cycled in the following cycles, and a comparable cycling behavior is observed. In strong contrast to this, for recycled LFP, no considerable redox activity is observed during cycling, suggesting significant degradation of the active electrode material, though no strong compositional changes were detected. It is important to note that pristine LFP already exhibits only comparatively low currents, which might be related to the impurity phases present in the sample and the lack of carbon coating.

Aside from LMO, the most significant changes in cycling behavior are observed with recycled NMC811 and LFP. For these two electrode materials, it is worthwhile to take a closer look at potential degradation reactions. Given the relatively low solubility of the investigated electrode materials in EtOH, it is likely that reactions predominantly occur between the electrolyte ions in solution and the surfaces of the electrode particles. Such surface reactions are crucial as they can result in the formation of protective layers on the particles. For example, modifying electrode materials with sulfur, polysulfides or Li_2_S additives has been shown to enhance electrochemical performance.[^62^, ^63^, ^64^] However, it is also possible that such modifications could lead to the formation of unfavorable passivating layers.

To gain insights into such surface reactions and potential morphology differences after the solution‐based separation process, pristine and recycled NMC811 and LFP were examined using SE microscopy (Figure S 10 and S 11) and EDX. No clear distinction in the shape or size of particles between the pristine and recycled materials is observed. However, during the measurement of recycled LFP separated from Li_6_PS_5_Br, significantly less surface charging was observed. This could indicate that an electronically conducting surface layer may have formed on the particles during the dissolution process. Since the S, Cl, Br and I concentrations of the recycled electrode materials could not be determined by ICP‐MS, EDX (Table S 9) was used for their analysis. The stoichiometry of the electrode materials was confirmed, with a more precise quantification achieved using ICP‐MS (compare values in Table 9). Within the error of EDX, depending on the used SE, varying amounts of impurity elements (P, S, Cl, Br and I) are detected, with some samples showing impurities of more than 12 at.%. This clearly indicates that carryover of these elements into the electrode material fraction occurred.

To qualitatively investigate differences of surface compositions of the electrode materials before and after the dissolution‐based separation process, XPS measurements were performed. Due to the close proximity, partial overlap and complex splitting of the peaks of different metal species (e. g., Ni^2+^‐O, Ni^2+^‐Cl, Ni^2+^‐Br, Ni^2+^‐I, Ni^2+^‐S, Ni^3+^‐O), a quantitative analysis was omitted. Instead, the discussion is based on qualitative differences between the spectra.

**Figure 9:**
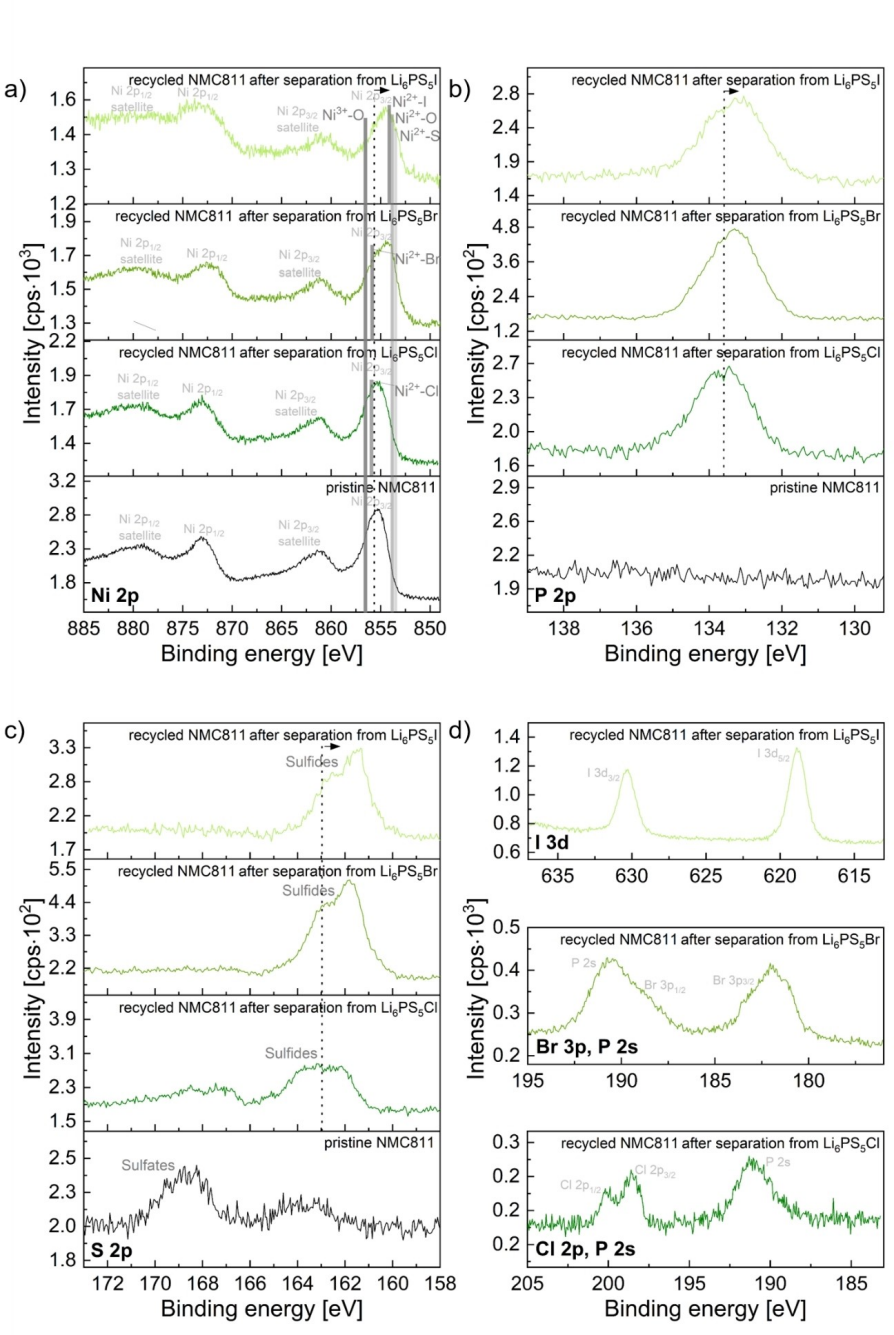
Ni 2p (a), P 2p (b), S 2p (c) and Cl 2p/P 2 s, Br 3p/P 2 s, I 3 d (d) XP spectra of recycled NMC811 after separation from Li_6_PS_5_Cl, Li_6_PS_5_Br, and Li_6_PS_5_I in comparison to pristine NMC811. For the dissolution of the SEs, EtOH was used (solid:liquid ratio of 10 mg:1 ml, stirring time of 24 h). In a), the different species (e. g., Ni^2+^‐O, Ni^3+^‐O) are indicated at their expected binding energies in dark grey. It should be noted that the species are only indicated at the Ni 2p_3/2_ main feature. The dashed black lines mark the centers of gravity of the respective signals, highlighting the shifts and broadening of the different spectra in relation to each other.

A comparison of the Ni 2p, P 2p, and S 2p XP spectra of the pristine and recycled NMC811 is given in Figure 9; the characteristic spectra of the halides are shown as well. Additional spectra (e. g., O 1s and C1s) are provided in Figure S 12. Since Ni is the main transition metal in NMC811, it is interesting to examine differences in the Ni 2p spectra between the pristine and recycled materials. It becomes obvious that all three recycled materials show significant shifts towards lower binding energies, which is indicated in Figure 9 a by the shift of the center of gravity of the Ni 2p_3/2_ signal. In addition, a broadening of the peak is observed. This shift and broadening are due to the presence of various Ni species on the surface of recycled NMC811. The binding energies of these species can be ordered as follows: Ni^3+^‐O > Ni^2+^‐Cl > Ni^2+^‐Br > Ni^2+^‐I > Ni^2+^‐O > Ni^2+^‐S.[^65^, ^66^] The expected binding energies of these species are indicated for the p_3/2_ main feature in the Ni 2p spectra. The observed shifts (indicated by the dashed line) and broadening vary depending on the species present and their relative amounts. Notably, a significant fraction of Ni^2+^‐containing species is detected, suggesting that the surface has a lower oxidation state compared to the bulk material.

The P 2p spectra of pristine and recycled NMC811 (Figure 9 b) show significant differences. As expected, there is no P 2p signal detected for the pristine material. Only after the separation from the SEs, phosphorous species are present. The comparatively high binding energy of this signal suggests a considerable formation of P–[S]_n_–P bonds and oxygenated phosphorus species such as phosphates, metaphosphates, or Li_3_PS_4‐x_O_x_.[^67^, ^68^] In contrast to this, the binding energy of P‐S bonds in ortho‐thiophosphates PS_4_^3−^ units, as found in argyrodite‐type electrolytes, is reported to be lower (~132 eV)[^69^, ^70^, ^71^]. This indicates that it is unlikely that high fractions of PS_4_^3−^‐containing phases are present on the particle surfaces. This is particularly pronounced for NMC811 separated from Li_6_PS_5_Cl, showing the highest shift towards higher binding energies (compare the shifts of the P 2p signals in relation to the dashed line which marks the center of gravity of NMC811 separated from Li_6_PS_5_Cl). Note, that the C‐O peaks of C 1s and O 1s peaks (Figure S 12) are not shifted in binding energy. Therefore, it can be excluded that the shift is due to different amounts of surface charging or an error in binding energy calibration.

The S 2p spectra (Figure 9 c) reveal the presence of sulfidic species in all four samples. Interestingly, the spectrum of pristine NMC811 features unexpectedly a signal at binding energies characteristic of metal sulfates.^[72]^ This might be related to reactions between NMC811 and gaseous sulfur species during prolonged storage in the glovebox which contains a variety of sulfur‐containing compounds. After the separation process, these metal sulfates are significantly reduced or completely removed from the recycled particles. Instead, the recycled materials show peaks that can be attributed to metal sulfides. Similar to the P 2p spectra of recycled NMC811, the center of gravity of the S 2p signals is found to be at higher binding energies in the sample separated from Li_6_PS_5_Cl and shifts towards lower energies for the Li_6_PS_5_Br and Li_6_PS_5_I samples. This agrees with an increased presence of P‐[S]_n_‐P type bonds (n=2: 162.7 eV) and oxygenated species.^[73]^ In addition, other sulfidic species such as for example NiS (~162.4 eV)[^74^, ^75^], PS_4_^3−^ in Li_6_PS_5_X (161.7 eV)[^70^, ^73^] or Li_2_S (~159.8 eV)[^70^, ^73^] are typically found at these lower energies. The presence of these different species impedes deconvolution.

Finally, the presence of the halide‐containing phases on the surface of the recycled electrode materials could be confirmed by XPS (Figure 9 d). The detected Cl 2p doublet corresponds well to NiCl_2_ (Ni 3p_3/2_: ~198.3 eV)^[76]^, the Br 3p_1/2_ and 3p_3/2_ signals align with NiBr_2_ (Br 3p_3/2_: ~182.0 eV)^[77]^, and the signals in the I 3 d spectrum can be attributed to NiI_2_ (I 3d_5/2_: ~619.0 eV)^[78]^. These species are not found in pristine NMC811 (not shown).

**Figure 10:**
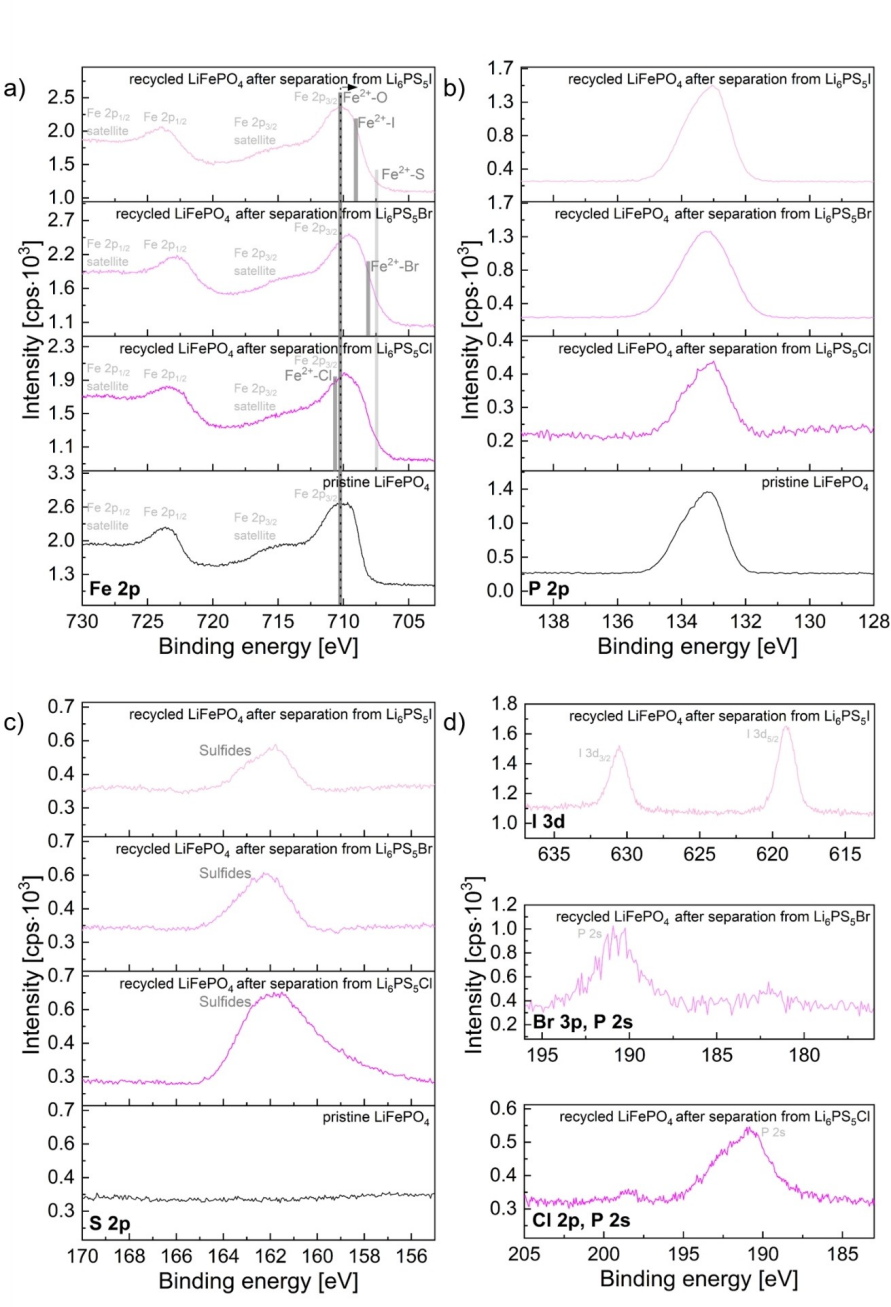
Fe 2p (a), P 2p (b), S 2p (c) and Cl 2p/P 2 s, Br 3p/P 2 s, I 3 d (d) XP spectra of recycled LiFePO_4_ after separation from Li_6_PS_5_Cl, Li_6_PS_5_Br, and Li_6_PS_5_I in comparison to pristine LiFePO_4_. For the dissolution of the SEs, EtOH was used (solid:liquid ratio of 10 mg:1 ml, stirring time of 24 h). In a), The binding energies of different species (e. g., Fe^2+^‐O, Fe^2+^‐S) are indicated at their expected binding energies in dark grey. It should be noted that the species are only indicated at the Fe 2p_3/2_ main feature. The dashed black line marks the center of gravity of the Fe 2p_3/2_ signal, highlighting the shifts and broadening of the different spectra in relation to each other.

Similar observations can be made in pristine and recycled LFP (Figure 10 and Figure S 13). However, when comparing the Fe 2p spectra of LFP and the Ni 2p spectra of NMC811, less pronounced shifts to lower binding energies are found for the recycled LFP. This agrees with the fact that Fe in LFP possesses an oxidation state of +2. Nevertheless, a shoulder towards lower energies is present which suggests that again other Fe^2+^‐containing phases (e. g., FeCl_2_, FeBr_2_, FeI_2_, FeS/FeS_2_)[^79^, ^80^] are formed. The P 2p signals of all samples are very similar and confirm the predominant presence of phosphate species PO_4_^3−^ of LiFePO_4_.[^81^, ^82^] The S 2p spectrum of recycled LFP confirms again the formation of sulfidic species on the particle surfaces.[^69^, ^80^, ^83^, ^84^] In agreement with EDX measurements (Table S 9), the Cl 2p and Br 3 d spectra give little to no indication for the presence of FeCl_2_ or FeBr_2_. This suggests that changes in the Fe 2p spectra are predominantly due to sulfidic species. In contrast to this, FeI_2_ signals can be found in the I 3 d spectrum.

## Conclusions

This study underscores the complexities and potential of recycling ASSBs through a dissolution‐based separation strategy. Our investigation reveals that the choice of solvent, and concentration of electrolyte significantly influence the recrystallization behavior of argyrodite thiophosphate electrolytes. The solvent should possess the ability to fully dissolve the SEs at reasonable high concentrations while minimizing nucleophilic attacks as much as possible. Although EtOH is a commonly used solvent for SE processing, alternative, less nucleophilic solvents (e. g., NMF) could provide the benefit of reduced degradation of the SEs.

Additionally, we demonstrate that interactions between dissolved electrolytes and transition metal oxide electrode materials can lead to significant chemical reactions, affecting the functional properties of both components. A dependence of the degradation mechanism based on the electrode material is observed, with the most pronounced impact occurring when separating the SEs from NMC811 and LFP.

The findings highlight the necessity for careful consideration of these degradation reactions in the material selection and design process of ASSBs. The structural and compositional analyses provide an understanding that can improve the development of efficient and sustainable recycling methods for ASSBs, ultimately contributing to the advancement of next‐generation battery technologies.

For future applications, the influence of metallic lithium from the anode remains a critical factor that must be addressed to ensure the viability of this recycling. The nucleophilicity of solvents used will play a crucial role and warrants further investigation. Additionally, the effectiveness of the recycling strategy will depend on the amount of residual lithium, which is determined by factors such as the battery′s state of discharge, design (e. g., lithium foil thickness), and the potential for anode‐free configurations. These variables highlight the need for targeted studies to assess the method′s applicability and efficiency under different conditions.

## Author Contributions

KW and OC conceived and designed the study. KW, ZH and XW prepared the samples and performed measurements. KW analyzed and interpreted the data and wrote the manuscript. MJ performed SEM and EDX measurements. KK performed XPS measurements on the electrode materials together with US. All authors discussed and revised the work.

## Conflict of Interests

There are no conflicts of interest to declare.

1

## Supporting information

As a service to our authors and readers, this journal provides supporting information supplied by the authors. Such materials are peer reviewed and may be re‐organized for online delivery, but are not copy‐edited or typeset. Technical support issues arising from supporting information (other than missing files) should be addressed to the authors.

Supporting Information

## Data Availability

The data that support the findings of this study are available from the corresponding author upon reasonable request.
