# Supplementary material for: Towards Recycling of All‐Solid‐State Batteries with Argyrodite Sulfide Electrolytes: Insights into Electrolyte and Electrode Degradation in Dissolution‐Based Separation Processes
Source: ChemSusChem. 2025 Jan 20;18(9):e202402128. doi: 10.1002/cssc.202402128 (PMC12051256; doi:10.1002/cssc.202402128)
Supplement: Supplementary file 1 — Supporting Information [file CSSC-18-e202402128-s001.pdf]

ChemSusChem

Supporting Information

Towards Recycling of All-Solid-State Batteries with Argyrodite Sulfide Electrolytes: Insights into Electrolyte and Electrode Degradation in Dissolution-Based Separation Processes

Kerstin Wissel,* Zian Hu, Xuebin Wu, Martine Jacob, Kathrin Küster, Ulrich Starke, and Oliver Clemens

Supplementary Information

Towards Recycling of All-Solid-State Batteries with Argyrodite Sulfide Electrolytes: Insights into Electrolyte and Electrode Degradation in Dissolution-Based Separation Processes

Kerstin Wissel[a,*], Zian Hu[a], Xuebin Wu[a], Martine Jacob[a], Kathrin Küster[b], Ulrich Starke[b], and Oliver Clemens[a, c]

[a] University of Stuttgart, Institute for Materials Science, Chemical Materials Synthesis, Heisenbergstraβe 3, 70569 Stuttgart, Germany

[b] Max Planck Institute for Solid State Research, Heisenbergstraße 1, 70569 Stuttgart, Germany

[c] Technical University of Darmstadt, Institute for Materials Science, Materials Analysis, Alarich-Weiss-Straße 2, 64287 Darmstadt, Germany

Corresponding Author:

Dr. Kerstin Wissel

E-Mail: kerstin.wissel@imw.uni-stuttgart.de

Fax: +49 711 685 61963

Table S 1: Physical and chemical properties of solvents used in this study. [1]In addition, the ability of the solvent to completely dissolve 10 mg of Li_6PS_5Cl in 1 ml of solvent is indicated.

Solvent	Classification	Dielectric contant ε_r	Polarity index	Boiling point T_{bp} [°C]	Complete dissolution of 10 mg SE in 1 ml solvent?
Hexane	Non-polar	1.88	0.009	68.7	No
Tetrahydrofuran (THF)	Aprotic, polar	7.58	0.207	66.0	No
Ethyl acetate (EA)	Aprotic, polar	6.02	0.228	77.2	No
Acetonitril (ACN)	Aprotic, polar	35.94	0.460	81.6	No
N-Methylformamide (NMF)	Protic, polar	182.40	0.722	200	Yes
Methanol (MeOH)	Protic, polar	32.66	0.762	64.5	Yes
Ethanol (EtOH)	Protic, polar	24.55	0.654	78.3	Yes
Isoproponal (i-PrOH)	Protic, polar	19.92	0.546	82.2	No
Tert-Butanol (t-BuOH)	Protic, polar	12.47	0.389	82.3	No

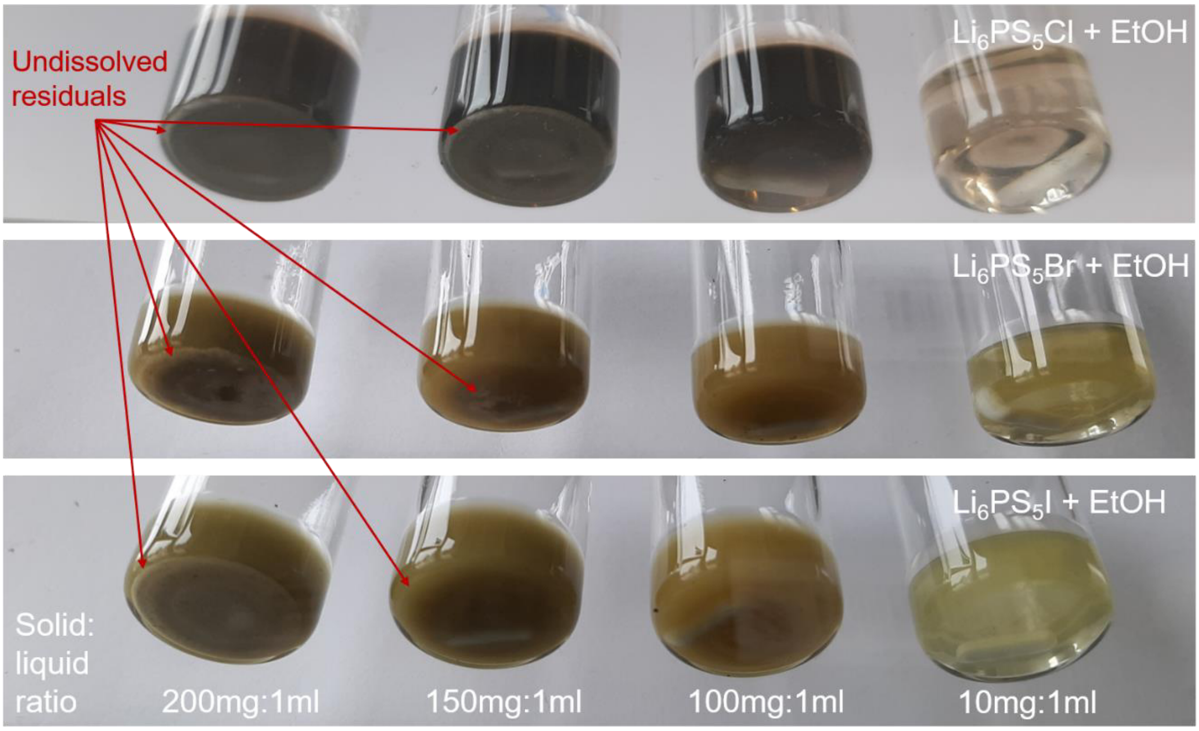

Figure S 1: Photograph of Li_6PS_5X (C = Cl, Br, I) in EtOH when using different solid-to-liquid ratios of Li_6PS_5Cl to EtOH.

Table S 2: Quantitative analysis and refined lattice parameters of pristine and recrystallized Li_6PS_5Cl after the dissolution using different solid:liquid ratios of Li_6PS_5Cl to EtOH and reaction times. The samples were heated to 550 °C for 10 h in evacuated quartz ampoules.

	**Phase**	**Phase fraction [wt.%]**	**Space group**	**a [Å]**	**b [Å]**	**c [Å]**	**Volume [Å^3]**
**pristine Li_6PS_5Cl**	Li_6PS_5Cl	100	*F*-43*m*	9.84681(3)			954.74(2)
**200mg:1ml, 1h**	Li_6PS_5Cl	81.7(12)	*F*-43*m*	9.85040(9)			955.79(3)
	Li_2S	2.1(1)	*Fm*-3*m*	5.7091(3)			186.08(3)
	LiCl	11.7(12)	*Fm*-3*m*	5.1447(3)			136.17(3)
	Li_3PO_4	4.5(3)	*Pnma*	10.531(7)	6.125(5)	4.924(3)	317.6(4)
**200mg:1ml, 24h**	Li_6PS_5Cl	83.3(6)	*F*-43*m*	9.8496(1)			955.64(3)
	Li_2S	3.3(2)	*Fm*-3*m*	5.7043(6)			185.65(6)
	LiCl	8.8(6)	*Fm*-3*m*	5.1445(4)			136.16(3)
	Li_3PO_4	5.1(4)	*Pnma*	10.544(8)	6.118(5)	4.923(3)	317.6(4)
**100mg:1ml, 1h**	Li_6PS_5Cl	75.9(3)	*F*-43*m*	9.85278(9)			956.48(3)
	Li_2S	6.73(10)	*Fm*-3*m*	5.7103(1)			186.20(1)
	LiCl	5.57(11)	*Fm*-3*m*	5.1437(2)			136.09(1)
	Li_3PO_4	11.8(3)	*Pnma*	10.519(3)	6.122(2)	4.923(1)	317.07(15)
**100mg:1ml, 24h**	Li_6PS_5Cl	72.8(2)	*F*-43*m*	9.85336(8)			956.65(2)
	Li_2S	8.48(8)	*Fm*-3*m*	5.71049(9)			186.217(9)
	LiCl	6.42(9)	*Fm*-3*m*	5.1461(2)			136.28(1)
	Li_3PO_4	12.3(2)	*Pnma*	10.522(2)	6.1195(12)	4.9224(7)	316.95(10)
**10mg:1ml, 1h**	Li_6PS_5Cl	64.7(3)	*F*-43*m*	9.8707(2)			961.70(5)
	Li_2S	7.1(1)	*Fm*-3*m*	5.7130(2)			186.46(2)
	LiCl	11.8(2)	*Fm*-3*m*	5.1476(2)			136.40(1)
	Li_3PO_4	16.4(2)	*Pnma*	10.501(1)	6.1192(6)	4.9223(4)	316.28(5)
**10mg:1ml, 24h**	Li_6PS_5Cl	62.2(3)	*F*-43*m*	9.8526(1)			956.41(4)
	Li_2S	11.0(1)	*Fm*-3*m*	5.7107(1)			186.24(1)
	LiCl	9.6(1)	*Fm*-3*m*	5.1445(2)			136.15(1)
	Li_3PO_4	17.2(3)	*Pnma*	10.507(2)	6.1209(12)	4.9231(8)	316.62(10)

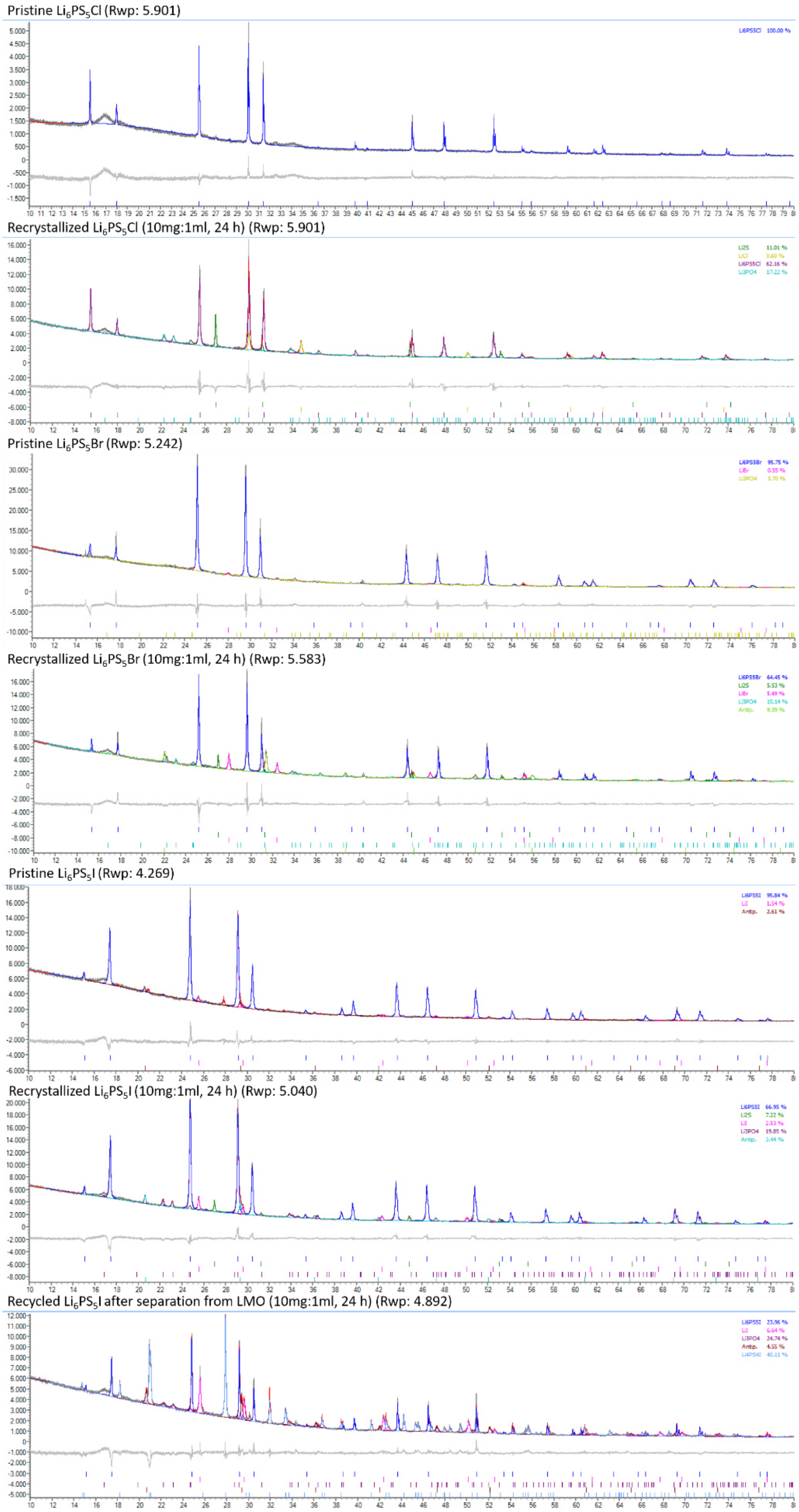

Figure S 2: Exemplary Rietveld refinements of pristine, recrystallized and recycled SEs. The measured data is depicted in dark grey, the calculated curve in red, the difference curve in light grey and the curves of the phases present in the color indicated in the legend.

Table S 3: Ratios of PS_4 to OH for different concentrations of Li_6PS_5Cl and alcohols. The values of the conducted experiments are marked in gray.

	**Ratio of PS_4 to OH in 1ml MeOH**	**Ratio of PS_4 to OH in 1ml EtOH**	**Ratio of PS_4 to OH in in 1ml i-PrOH**	**Ratio of PS_4 to OH in in 1ml t-BuOH**
**200 mg Li_6PS_5Cl**	$7.45 \cdot 10^{-4}$ mol/ 0.0247 mol	$7.45 \cdot 10^{-4}$ mol/ 0.0171 mol	$7.45 \cdot 10^{-4}$ mol/ 0.0131mol	$3.73 \cdot 10^{-4}$ mol/ 0.0104 mol
**100 mg Li_6PS_5Cl**	$3.73 \cdot 10^{-4}$ mol/ 0.0247mol	$3.73 \cdot 10^{-4}$ mol/0.0171 mol	$3.73 \cdot 10^{-4}$ mol/0.0131 mol	$3.73 \cdot 10^{-4}$ mol/ 0.0104 mol
**10 mg Li_6PS_5Cl**	$3.73 \cdot 10^{-5}$ mol/ 0.0247mol	$3.73 \cdot 10^{-5}$ mol/ 0.0171 mol	$3.73 \cdot 10^{-5}$ mol/ 0.0131 mol	$3.73 \cdot 10^{-5}$ mol/ 0.0104 mol

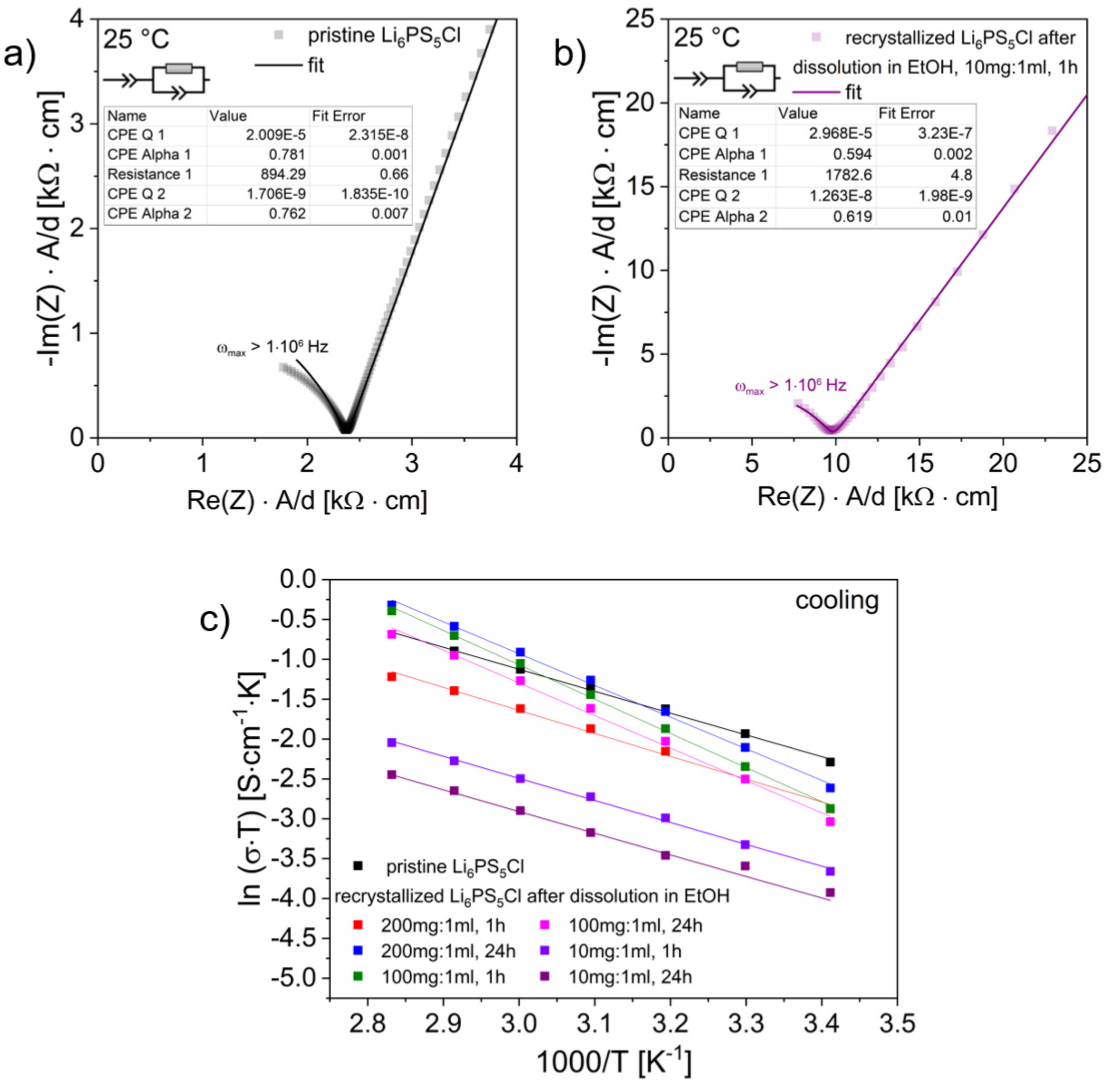

Figure S 3: Representative Nyquist plots with corresponding fits and fitted parameters of pristine Li_6PS_5Cl (a) and recrystallized Li_6PS_5Cl after dissolution in EtOH (10mg:1ml, 1h) (b). Arrhenius plots of pristine and recrystallized Li_6PS_5Cl after the dissolution using different solid:liquid ratios of Li_6PS_5Cl to EtOH and reaction times (c). The capacitance C was calculated according to $C = R^{\left(\frac{1-\alpha}{\alpha}\right)} \cdot Q^{\frac{1}{\alpha}}$.

Table S 4: Quantitative analysis and refined lattice parameters of pristine, and recrystallized Li_6PS_5Cl after the dissolution in MeOH, EtOH, i-PrOH, and t-BuOH (solid:liquid ratio of 10mg:1ml, stirring time of 24 h). The samples were heated to 550 °C for 10 h in evacuated quartz ampoules.

	**Phase**	**Phase fraction [wt.%]**	**Space group**	**a [Å]**	**b [Å]**	**c [Å]**	**Volume [Å^3]**
**pristine Li_6PS_5Cl**	Li_6PS_5Cl	100	*F*-43*m*	9.84681(3)			954.74(2)
**recrystallized Li_6PS_5Cl after dissolution in different alcohols (10 mg:1 ml, 24h)**							
**MeOH**	Li_6PS_5Cl	26.0(3)	*F*-43*m*	9.9224(18)			976.90(1)
	Li_2S	14.95(18)	*Fm*-3*m*	5.7253(10)			187.68(3)
	LiCl	16.27(19)	*Fm*-3*m*	5.1583(9)			137.25(1)
	Li_3PO_4	42.7(3)	*Pnma*	10.5185(19)	6.1348(11)	4.9356(9)	318.49(10)
**EtOH**	Li_6PS_5Cl	62.2(3)	*F*-43*m*	9.8526(1)			956.41(4)
	Li_2S	11.0(1)	*Fm*-3*m*	5.7107(1)			186.24(1)
	LiCl	9.6(1)	*Fm*-3*m*	5.1445(2)			136.15(1)
	Li_3PO_4	17.2(3)	*Pnma*	10.507(2)	6.1209(12)	4.9231(8)	316.62(10)
**i-PrOH**	Li_6PS_5Cl	67.0(2)	*F*-43*m*	9.8787(19)			963.84(1)
	Li_2S	5.75(9)	*Fm*-3*m*	5.7216(11)			187.31(2)
	LiCl	7.95(9)	*Fm*-3*m*	5.1525(10)			136.79(4)
	Li_3PO_4	19.3(2)	*Pnma*	10.519(3)	6.122(2)	4.923(1)	317.02(1)
**t-BuOH**	Li_6PS_5Cl	80.9(3)	*F*-43*m*	9.892(2)			967.95(2)
	Li_2S	1.3(2)	*Fm*-3*m*	5.715(2)			185.66(1)
	LiCl	14.3(1)	*Fm*-3*m*	5.1974(12)			140.40(5)
	Li_3PO_4	3.5(4)	*Pnma*	10.498(9)	6.134(5)	4.940(4)	318.10(1)

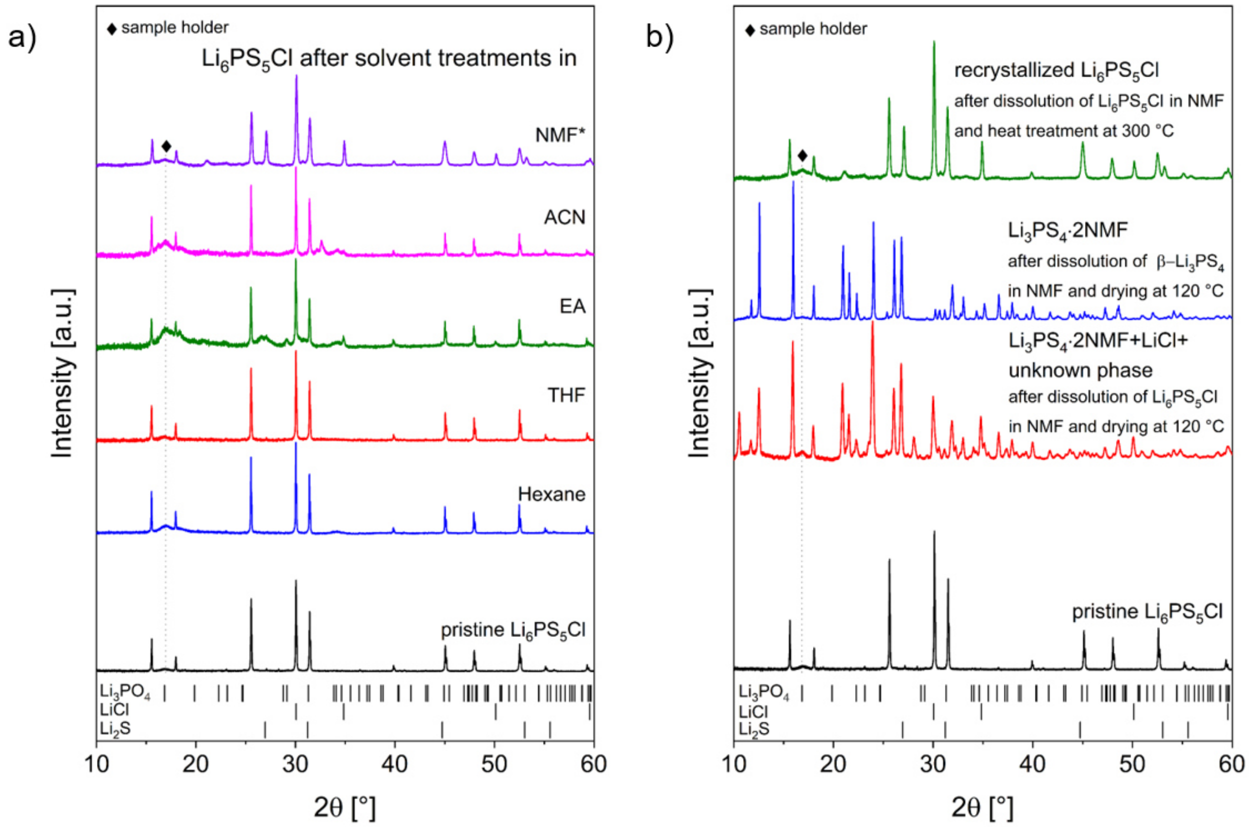

*Figure S 4: a) X-ray diffraction patterns of precipitates obtained after solvent treatment of Li_6PS_5Cl in different organic solvents in comparison to pristine Li_6PS_5Cl. To remove the solvents, the samples were heated to 120 °C for 2 h at reduced pressures. To decompose the formed intermediate NMF solvent complex (see also Figure S 4 b), the NMF sample marked with * was additionally heated to 300 °C for 2 h at a reduced pressure. b) X-ray diffraction patterns of precipitates obtained after solvent treatment of Li_6PS_5Cl in NMF and drying at 120 °C in comparison to $Li_3PS_4 \cdot 2NMF$. The dissolution leads to the formation of $Li_3PS_4 \cdot 2NMF$, LiCl and an unknown phase. After the additional heat treatment at 300 °C, recrystallized Li_6PS_5Cl is found. The positions of the characteristic reflection ticks of decomposition products Li_2S, LiCl, and Li_3PO_4 observed after the dissolution and recrystallization are given.*

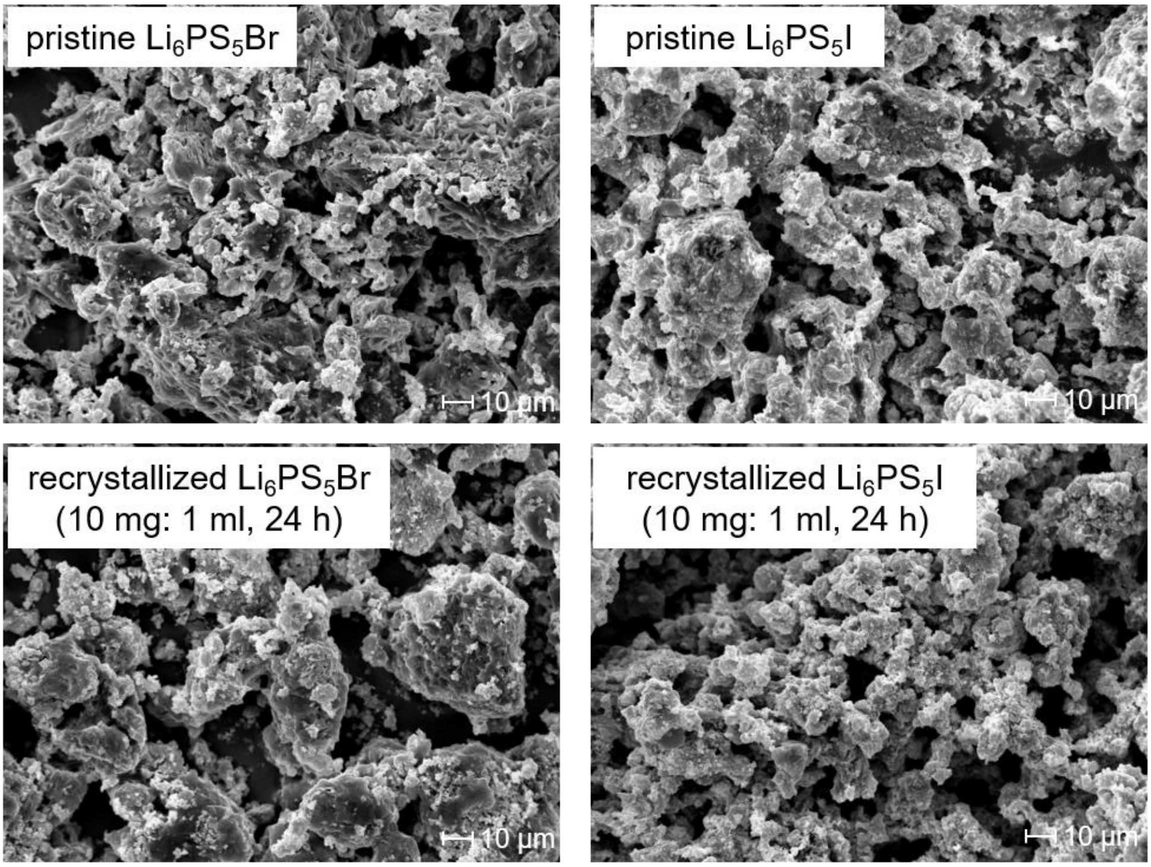

Figure S 5: SE micrographs of pristine and recrystallized Li_6PS_5Br and Li_6PS_5I after the dissolution in EtOH (solid:liquid ratio of 10 mg:1 ml, stirring time of 24 h). The recrystallized samples were heated to 550 °C for 10 h in evacuated quartz ampoules.

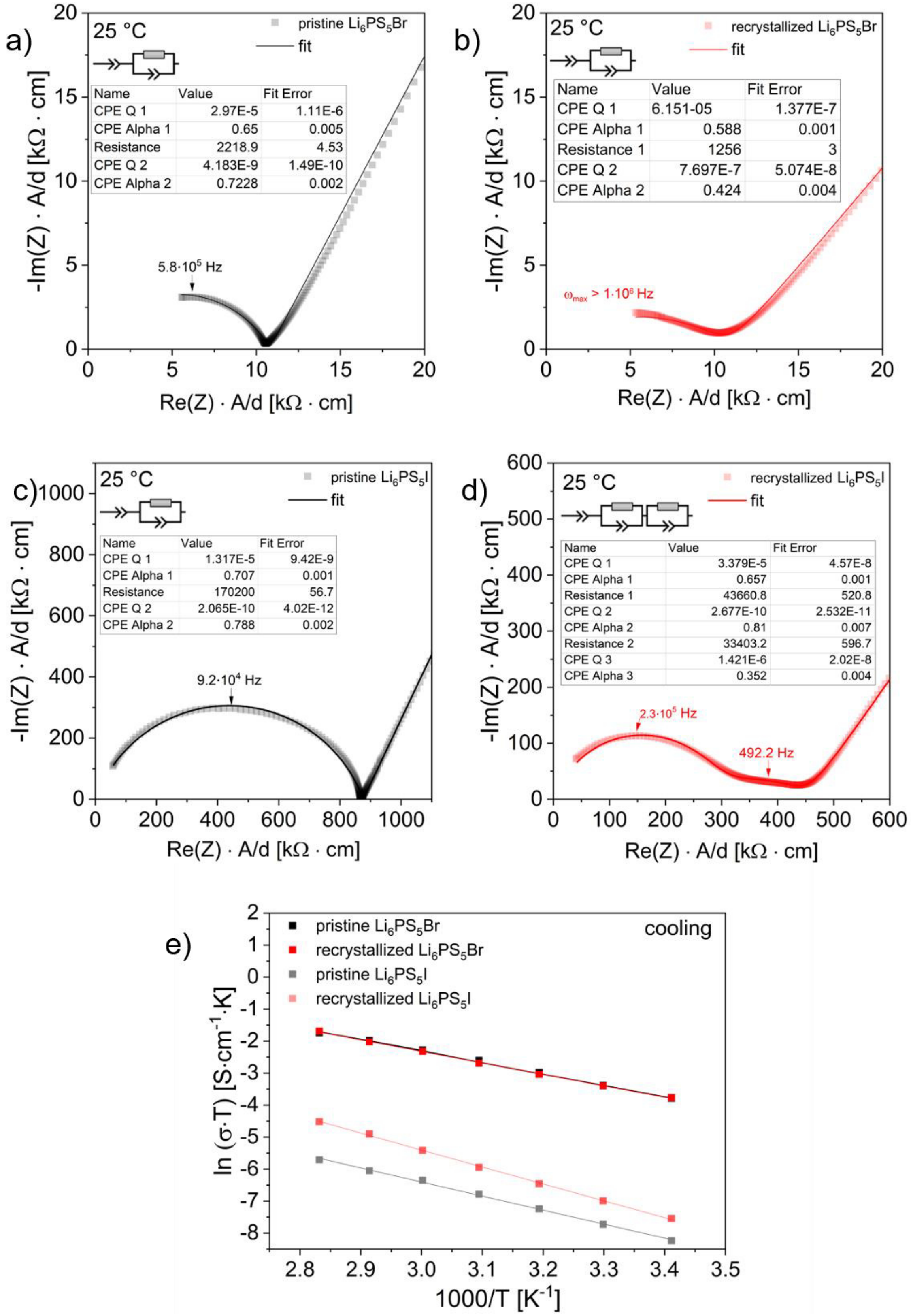

Figure S 6: Nyquist plots with corresponding fits and fitted parameters of pristine Li_6PS_5Br (a), recrystallized Li_6PS_5Br after dissolution in EtOH (10mg:1ml, 24h) (b), pristine Li_6PS_5I (c), and recrystallized Li_6PS_5I after dissolution in EtOH (10mg:1ml, 24h) (d). Arrhenius plots of pristine and recrystallized Li_6PS_5Br and Li_6PS_5I after the dissolution in EtOH (solid:liquid ratio of 10mg:1ml, stirring time of 24 h) (e). The capacitance C was calculated according to $C = R^{\left(\frac{1-\alpha}{\alpha}\right)} \cdot Q^{\frac{1}{\alpha}}$.

Table S 5: Quantitative analysis and refined lattice parameters of pristine, recrystallized and recycled Li_6PS_5Cl after the dissolution in EtOH (solid:liquid ratio of 10mg:1ml, stirring time of 24 h) and separation from different electrode materials. The samples were heated to 550 °C for 10 h in evacuated quartz ampoules.

	Phase	Phase fraction [wt.%]	Space group	a [Å]	b [Å]	c [Å]	Volume [Å^3]
**pristine Li_6PS_5Cl**	Li_6PS_5Cl	100	*F*-43*m*	9.84681(3)			954.74(2)
**recrystallized Li_6PS_5Cl (10mg:1ml, 24 h, no electrode material)**	Li_6PS_5Cl	62.2(3)	*F*-43*m*	9.8526(1)			956.41(4)
	Li_2S	11.0(1)	*Fm*-3*m*	5.7107(1)			186.24(1)
	LiCl	9.6(1)	*Fm*-3*m*	5.1445(2)			136.15(1)
	Li_3PO_4	17.2(3)	*Pnma*	10.507(2)	6.1209(12)	4.9231(8)	316.62(10)
**recrystallized Li_6PS_5Cl after dissolution in EtOH (10 mg:1 ml, 24 h) and separation from**							
**$LiCoO_2$**	Li_6PS_5Cl	55.6(4)	*F*-43*m*	9.8706(3)			961.68(7)
	Li_2S	11.1(2)	*Fm*-3*m*	5.71237(13)			186.40(1)
	LiCl	9.7(2)	*Fm*-3*m*	5.14703(14)			136.35(1)
	Li_3PO_4	23.6(4)	*Pnma*	10.5012(15)	6.1225(9)	4.9263(6)	316.73(7)
**$LiMn_2O_4$**	Li_6PS_5Cl	73.2(3)	*F*-43*m*	9.83342(13)			950.86(4)
	Li_2S	-	*Fm*-3*m*	-			-
	LiCl	5.5(1)	*Fm*-3*m*	5.1496(3)			136.56(2)
	Li_3PO_4	21.3(3)	*Pnma*	10.5219(13)	6.1311(8)	4.9324(5)	318.19(6)
**NMC811**	Li_6PS_5Cl	40.2(2)	*F*-43*m*	9.85843(14)			958.12(4)
	Li_2S	15.23(13)	*Fm*-3*m*	5.71046(7)			186.21(1)
	LiCl	13.27(16)	*Fm*-3*m*	5.14328(8)			136.06(1)
	Li_3PO_4	31.3(2)	*Pnma*	10.5055(8)	6.1240(5)	4.9277(3)	317.03(4)
**$LiFePO_4$**	Li_6PS_5Cl	41.82(18)	*F*-43*m*	9.85770(12)			957.91(4)
	Li_2S	14.48(11)	*Fm*-3*m*	5.71037(6)			186.21(1)
	LiCl	14.90(15)	*Fm*-3*m*	5.14201(8)			135.96(1)
	Li_3PO_4	28.8(2)	*Pnma*	10.5039(8)	6.1222(4)	4.9258(3)	316.76(4)
**$Li_4Ti_5O_{12}$**	Li_6PS_5Cl	54.1(3)	*F*-43*m*	9.8655(2)			960.19(6)
	Li_2S	10.1(2)	*Fm*-3*m*	5.71006(12)			186.19(1)
	LiCl	15.3(3)	*Fm*-3*m*	5.14479(17)			136.18(1)
	Li_3PO_4	20.5(3)	*Pnma*	10.5027(12)	6.1206(7)	4.9232(5)	316.48(6)

Table S 6: Quantitative analysis and refined lattice parameters of pristine, recrystallized and recycled Li_6PS_5Br after the dissolution in EtOH (solid:liquid ratio of 10mg:1ml, stirring time of 24 h) and separation from different electrode materials. The samples were heated to 550 °C for 10 h in evacuated quartz ampoules.

	Phase	Phase fraction [wt.%]	Space group	a [Å]	b [Å]	c [Å]	Volume [Å^3]
**pristine Li_6PS_5Br**	Li_6PS_5Br	98.35(9)	*F*-43*m*	9.99921(14)			999.76(4)
	LiBr	0.55(9)	*Fm*-3*m*	5.5111(12)			167.39(11)
	Li_3PO_4	1.1(5)	*Pnma*	10.485(7)	6.122(4)	4.940(2)	317.1(3)
**recrystallized Li_6PS_5Br (10mg:1ml, 24h, no electrode material)**	Li_6PS_5Br	64.5(4)	*F*-43*m*	9.98538(10)			995.62(3)
	Li_2S	5.5(2)	*Fm*-3*m*	5.71614(13)			186.77(1)
	LiBr	5.5(1)	*Fm*-3*m*	5.51876(17)			168.08(2)
	Li_3PO_4	15.1(5)	*Pnma*	10.513(3)	6.1336(15)	4.935(1)	318.23(13)
	Li_3OBr/ $Li_2Br(OH)$/ $LiBr(H_2O)$	9.4(1)	*Pm*-3*m*	4.02630(11)			65.271(6)
**$LiCoO_2$**	Li_6PS_5Br	61.9(3)	*F*-43*m*	9.97598(9)			992.81(3)
	Li_2S	8.5(1)	*Fm*-3*m*	5.70922(10)			186.09(1)
	LiBr	12.5(1)	*Fm*-3*m*	5.49951(8)			166.330(7)
	Li_3PO_4	14.7(3)	*Pnma*	10.507(2)	6.1275(12)	4.9276(8)	317.22(11)
	Li_3OBr/ $Li_2Br(OH)$/ $LiBr(H_2O)$	2.4(1)	*Pm*-3*m*	4.0217(3)			65.05(1)
**$LiMn_2O_4$**	Li_6PS_5Br	68.7(5)	*F*-43*m*	9.9695(3)			990.88(8)
	Li_2S	-	*Fm*-3*m*	-			-
	LiBr	9.1(1)	*Fm*-3*m*	5.50309(19)			166.66(2)
	Li_3PO_4	22.2(5)	*Pnma*	10.517(3)	6.1382(17)	4.9324(1)	318.40(15)
	Li_3OBr/ $Li_2Br(OH)$/ $LiBr(H_2O)$	-	*Pm*-3*m*	-			-
**NMC811**	Li_6PS_5Br	61.7(4)	*F*-43*m*	9.98075(18)			994.24(5)
	Li_2S	4.8(2)	*Fm*-3*m*	5.7075(3)			185.93(3)
	LiBr	14.14(18)	*Fm*-3*m*	5.51898(15)			168.10(1)
	Li_3PO_4	17.6(5)	*Pnma*	10.502(3)	6.1243(17)	4.927(1)	316.90(2)

	Li_3OBr/ $Li_2Br(OH)$/ $LiBr(H_2O)$	1.76(12)	*Pm*-3*m*	4.0208(7)			65.00(4)
**$LiFePO_4$**	Li_6PS_5Br	53.1(3)	*F*-43*m*	9.96964(12)			990.89(4)
	Li_2S	9.10(14)	*Fm*-3*m*	5.71006(16)			186.175(15)
	LiBr	16.19(11)	*Fm*-3*m*	5.49995(8)			166.365(7)
	Li_3PO_4	19.7(4)	*Pnma*	10.518(2)	6.1288(12)	4.9286(8)	317.63(11)
	Li_3OBr/$Li_2Br(OH)$/ $LiBr(H_2O)$	1.91(7)	*Pm*-3*m*	4.0234(8)			65.13(1)
**$Li_4Ti_5O_{12}$**	Li_6PS_5Br	51.8(3)	*F*-43*m*	9.98574(12)			995.72(4)
	Li_2S	8.93(13)	*Fm*-3*m*	5.70910(12)			186.081(12)
	LiBr	16.74(12)	*Fm*-3*m*	5.49956(8)			166.335(7)
	Li_3PO_4	20.9(4)	*Pnma*	10.498(2)	6.1275(11)	4.9282(7)	317.01(9)
	Li_3OBr/$Li_2Br(OH)$/$LiBr(H_2O)$	1.63(8)	*Pm*-3*m*	4.0256(8)			65.23(1)

Table S 7: Quantitative analysis and refined lattice parameters of pristine, recrystallized and recycled Li_6PS_5I after the dissolution in EtOH (solid:liquid ratio of 10mg:1ml, stirring time of 24 h) and separation from different electrode materials. The samples were heated to 550 °C for 10 h in evacuated quartz ampoules.

	**Phase**	**Phase fraction [wt.%]**	**Space group**	**a [Å]**	**b [Å]**	**c [Å]**	**Volume [Å^3]**
**pristine Li_6PS_5I**	Li_6PS_5I	95.86(9)	*F*-43*m*	10.13699(9)			1041.66(3)
	LiI	1.52(7)	*Fm*-3*m*	6.0267(8)			218.90(8)
	$Li_3OI/Li_2I(OH)/LiI(H_2O)$	2.61(5)	*Pm*-3*m*	4.2955(2)			79.257(13)
**recrystallized Li_6PS_5I (10mg:1ml, 24h, no electrode material)**	Li_6PS_5I	66.9(3)	*F*-43*m*	10.15084(8)			1045.94(2)
	Li_2S	7.22(13)	*Fm*-3*m*	5.71420(15)			186.58(2)
	LiI	2.53(4)	*Fm*-3*m*	6.0310(2)			219.36(2)
	Li_3PO_4	19.9(4)	*Pnma*	10.4920(16)	6.1243(9)	4.9257(6)	316.51(8)
	$Li_3OI/Li_2I(OH)/LiI(H_2O)$	3.45(6)	*Pm*-3*m*	4.29653(15)			79.314(9)
**$LiCoO_2$**	Li_6PS_5I	68.8(5)	*F*-43*m*	10.14252(6)			1043.37(2)
	Li_2S	9.71(18)	*Fm*-3*m*	5.71027(10)			186.196(10)
	LiI	3.63(6)	*Fm*-3*m*	6.03382(15)			219.67(2)
	Li_3PO_4	16.5(6)	*Pnma*	10.511(4)	6.132(2)	4.930(2)	317.76(19)
	$Li_3OI/Li_2I(OH)/LiI(H_2O)$	1.36(7)	*Pm*-3*m*	4.2950(4)			79.23(2)
**$LiMn_2O_4$**	Li_6PS_5I	23.9(3)	*F*-43*m*		10.14292(8)		1043.49(2)
	Li_2S	-	*Fm*-3*m*	-			-
	LiI	6.64(9)	*Fm*-3*m*	6.02686(11)			218.91(1)
	Li_3PO_4	24.7(7)	*Pnma*	10.540(9)	6.125(5)	4.932(3)	318.4(4)
	$Li_3OI/Li_2I(OH)/LiI(H_2O)$	4.56(6)	*Pm*-3*m*	4.29592(11)			79.281(6)
	Li_4PS_4I	40.2(4)	*P4/nmm*	8.47918(10)		5.9293(1)	426.29(1)
**NMC811**	Li_6PS_5I	39.4(3)	*F*-43*m*	10.1654(2)			1050.46(5)
	Li_2S	9.79(17)	*Fm*-3*m*	5.71190(11)			186.36(1)
	LiI	9.46(9)	*Fm*-3*m*	6.03310(11)			219.59(1)
	Li_3PO_4	32.6(4)	*Pnma*	10.4925(10)	6.1231(6)	4.9265(4)	316.51(5)
	$Li_3OI/Li_2I(OH)/LiI(H_2O)$	8.75(9)	*Pm*-3*m*	4.29522(9)			79.242(5)

**$LiFePO_4$**	Li_6PS_5I	72.9(5)	*F*-43*m*	10.1447(1)			1044.05(3)
	Li_2S	6.0(2)	*Fm*-3*m*	5.7126(4)			186.43(4)
	LiI	4.80(7)	*Fm*-3*m*	6.03081(16)			219.345(18)
	Li_3PO_4	13.4(6)	*Pnma*	10.504(4)	6.130(2)	4.928(2)	317.3(2)
	$Li_3OI/Li_2I(OH)/LiI(H_2O)$	2.88 (8)	*Pm*-3*m*	4.2990(3)			79.447(17)
**$Li_4Ti_5O_{12}$**	Li_6PS_5I	61.3(3)	*F*-43*m*	10.14752(5)			1044.91(2)
	Li_2S	10.14(13)	*Fm*-3*m*	5.71170(6)			186.336(6)
	LiI	6.02(5)	*Fm*-3*m*	6.03345(7)			219.633(8)
	Li_3PO_4	21.1(4)	*Pnma*	10.506(2)	6.1349(12)	4.9292(8)	317.71(11)
	$Li_3OI/Li_2I(OH)/LiI(H_2O)$	1.44(5)	*Pm*-3*m*	4.2949(3)			79.225(17)

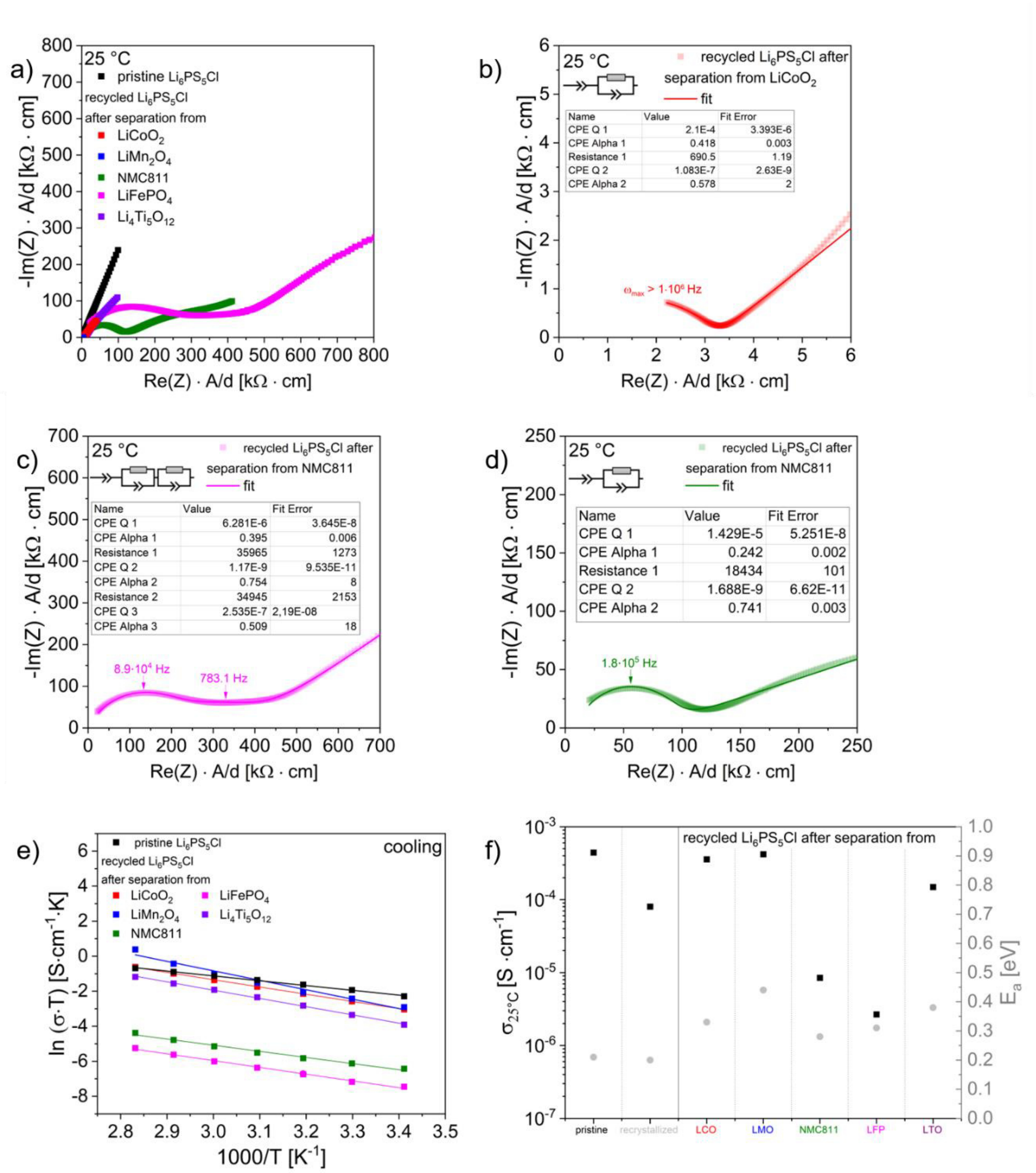

Figure S 7: Nyquist plots of recycled Li_6PS_5Cl after separation from electrode materials in comparison to pristine Li_6PS_5Cl (a); Nyquist plots with corresponding fits and fitted parameters of recycled Li_6PS_5Cl (b) after separation from $LiCoO_2$ (b), NMC811 (c) and $LiFEPO_4$ (d); Arrhenius plots of recycled Li_6PS_5Cl after separation from electrode materials (e); Ionic conductivities $\sigma_{25°C}$ and activation energies E_A of recycled Li_6PS_5Cl in comparison to pristine and recrystallized Li_6PS_5Cl (f). The capacitance C was calculated according to $C = R^{\left(\frac{1-\alpha}{\alpha}\right)} \cdot Q^{\frac{1}{\alpha}}$.

Table S 8: Quantitative analysis and refined lattice parameters of pristine and recycled electrode materials after separation from Li_6PS_5Cl, Li_6PS_5Br, and Li_6PS_5I. For the dissolution of the SEs, EtOH was used (solid:liquid ratio of 10mg:1ml, stirring time of 24 h).

	Phase	Phase fraction [wt.%]	Space group	a [Å]	b [Å]	c [Å]	α [°]	β [°]	γ [°]
**pristine $LiCoO_2$**	$LiCoO_2$	100	*R*-3*m*	2.81599(2)		14.0525(2)			
**recycled $LiCoO_2$ after separation from Li_6PS_5Cl**	$LiCoO_2$	100	*R*-3*m*	2.81773(4)		14.0676(3)			
**recycled $LiCoO_2$ after separation from Li_6PS_5Br**	$LiCoO_2$	100	*R*-3*m*	2.81613(4)		14.0614(3)			
**recycled $LiCoO_2$ after separation from Li_6PS_5I**	$LiCoO_2$	100	*R*-3*m*	2.81639(5)		14.0692(4)			
**pristine $LiMn_2O_4$**	$LiMn_2O_4$	100	*Fd*-3*m*	8.2410(2)					
**recycled $LiMn_2O_4$ after separation from Li_6PS_5Cl**	$Li_2Mn_2O_4$	100	$I4_1/amdZ$	5.6605(12)		9.250(2)			
**recycled $LiMn_2O_4$ after separation from Li_6PS_5Br**	$LiMn_2O_4$	19.8(7)	*Fd*-3*mZ*	8.253(3)					
	$Li_2Mn_2O_4$	80.2(7)	$I4_1/amdZ$	5.667(2)		9.261(3)			
**recycled $LiMn_2O_4$after separation from Li_6PS_5I**	$LiMn_2O_4$	15.0(6)	*Fd*-3*m*	8.234(2)					
	$Li_2Mn_2O_4$	84.8(4)	$I4_1/amdZ$	5.6599(11)		9.2798(18)			
**pristine NMC811**	NMC811	100	*R*-3*m*	2.87449(2)		14.2123(2)			
**recycled NMC811 after separation from Li_6PS_5Cl**	NMC811	100	*R*-3*m*	2.87462(3)		14.2107(3)			
**recycled NMC811 after separation from Li_6PS_5Br**	NMC811	100	*R*-3*m*	2.87660(3)		14.2176(4)			
**recycled NMC811 after separation from Li_6PS_5I**	NMC811	100	*R*-3*m*	2.87676(4)		14.2140(5)			
**pristine $LiFePO_4$**	$LiFePO_4$	88.2(6)	*Pnma*	10.3289(4)	6.0092(2)	4.6970(2)			
	$Li_4P_2O_7$	2.9(5)	$P12_1/n1$	5.305(7)	13.851(12)	8.050(14)		90.78(9)	
	$Fe_2P_2O_7$	2.1(3)	*P*-1	4.586(4)	5.215(6)	5.414(5)	102.22(6)	98.65(5)	100.46(5)
	Fe3O4	6.8(2)	*Fd*-3*mZ*	8.3713(10)					
**recycled $LiFePO_4$ after separation from Li_6PS_5Cl**	$LiFePO_4$	93.8(3)	*Pnma*	10.3330(3)	6.0125(2)	4.6983(1)			
	$Li_4P_2O_7$	1.2(2)	$P12_1/n1$	5.381(5)	13.841(11)	8.113(11)		91.07(10)	
	$Fe_2P_2O_7$	1.2(1)	*P*-1	4.4912(13)	5.2712(16)	5.5063(16)	103.85(2)	98.20(2)	99.02(2)
	Fe_3O_4	3.8(2)	*Fd*-3*mZ*	8.3714(5)					
**recycled $LiFePO_4$ after separation from Li_6PS_5Br**	$LiFePO_4$	88.8(5)	*Pnma*	10.3282(4)	6.0102(2)	4.69602(18)			
	$Li_4P_2O_7$	1.4(3)	$P12_1/n1$	5.312(4)	13.820(7)	8.126(6)		91.21(7)	
	$Fe_2P_2O_7$	0.86(18)	*P*-1	4.5448(18)	5.198(2)	5.481(2)	103.16(3)	98.37(3)	99.03(3)
	Fe_3O_4	9.0(4)	*Fd*-3*mZ*	8.3699(7)					
**recycled $LiFePO_4$ after separation from Li_6PS_5I**	$LiFePO_4$	91.9(4)	*Pnma*	10.3270(3)	6.0087(2)	4.6949(2)			
	$Li_4P_2O_7$	1.2(2)	$P12_1/n1$	5.305(3)	13.835(5)	8.088(4)		90.89(5)	
	$Fe_2P_2O_7$	1.8(2)	*P*-1	4.497(2)	5.256(3)	5.496(3)	103.67(4)	98.30(4)	99.04(4)
	Fe_3O_4	5.1(3)	*Fd*-3*mZ*	8.3693(11)					
**pristine $Li_4Ti_5O_{12}$**	$Li_4Ti_5O_{12}$	100	*Fd*-3*m*	8.36193(3)					

**recycled $Li_4Ti_5O_{12}$ after separation from Li_6PS_5Cl**	$Li_4Ti_5O_{12}$	100	*Fd*-3*m*	8.3638(7)
**recycled $Li_4Ti_5O_{12}$ after separation from Li_6PS_5Br**	$Li_4Ti_5O_{12}$	100	*Fd*-3*m*	8.3609(4)
**recycled $Li_4Ti_5O_{12}$ after separation from Li_6PS_5I**	$Li_4Ti_5O_{12}$	100	*Fd*-3*m*	8.3600(3)

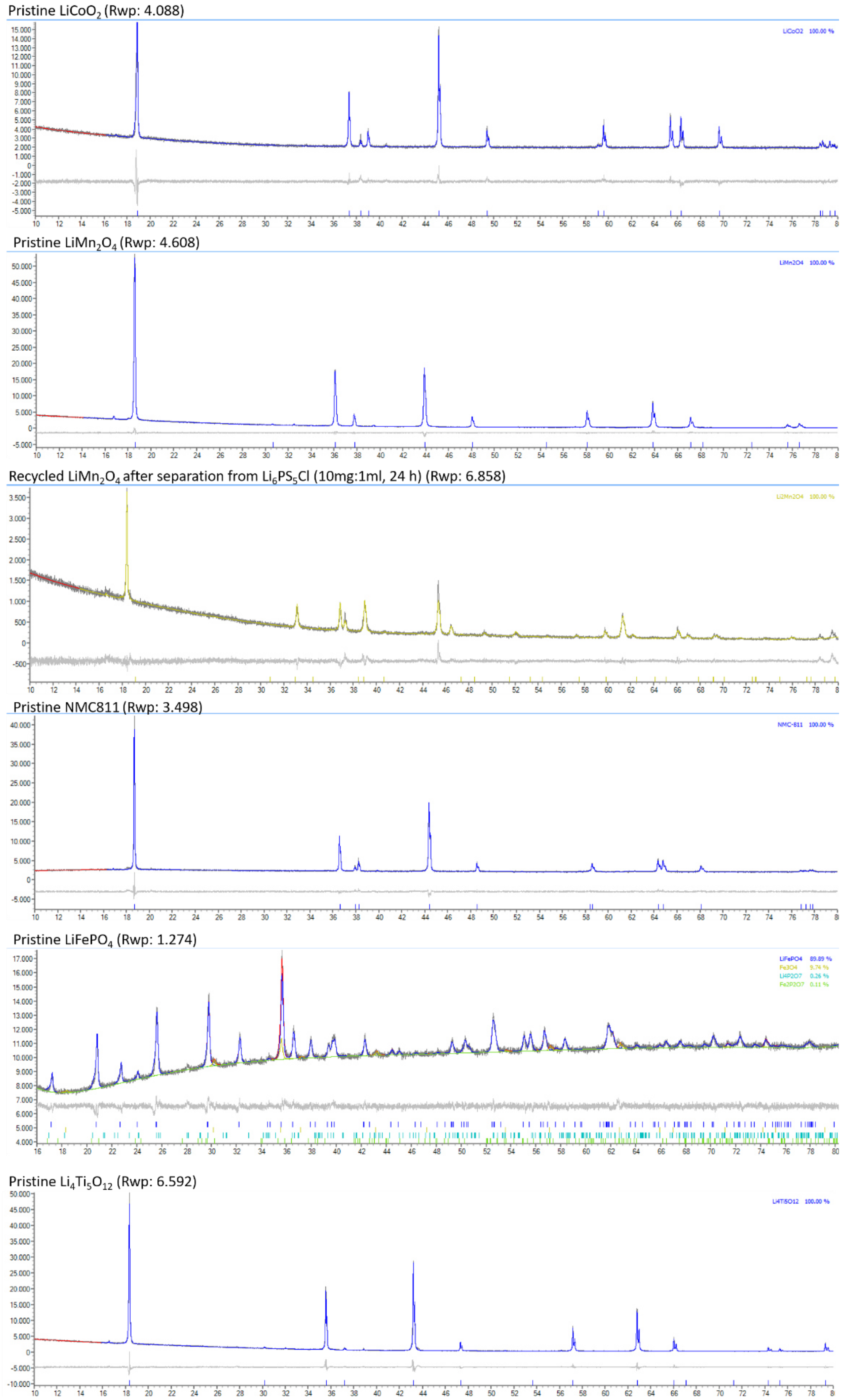

Figure S 8: Exemplary Rietveld refinements of pristine and recycled electrode materials after separation from Li_6PS_5Cl, Li_6PS_5Br, and Li_6PS_5I. For the dissolution of the SEs, EtOH was used (solid:liquid ratio of 10mg:1ml, stirring time of 24 h). The measured data is depicted in dark grey, the calculated curve in red, the difference curve in light grey and the curves of the phases present in the color indicated in the legend.

Table S 9: Comparison of compositions obtained from ICP-MS an EDX of pristine and recycled electrode materials after separation from after separation from Li_6PS_5Cl, Li_6PS_5Br, and Li_6PS_5I. For the dissolution of the SEs, EtOH was used (solid:liquid ratio of 10mg:1ml, stirring time of 24 h). ICP-MS measurements were not performed on $Li_4Ti_5O_{12}$ due to its high stability towards HCl used for dissolution of the electrode materials. EDX measurements were only performed on NMC811 and $LiFePO_4$.

	**Composition based on ICP-MS**	**EDX**									
		**At.% of Co [%]**	**At.% of Mn [%]**	**At.% of Ni [%]**	**At.% of Fe [%]**	**At.% of P [%]**	**At.% of S [%]**	**At.% of Cl [%]**	**At.% of Br [%]**	**At.% of I [%]**	**Composition based on EDX**
**pristine $LiCoO_2$**	$Li_{1.007}CoO_y$	-	-	-	-	-	-	-	-	-	-
**recycled $LiMn_2O_4$ after separation from $LiCoO_2$**	$Li_{1.262}CoO_y\,P_{0.006}$	-	-	-	-	-	-	-	-	-	-
**recycled $LiMn_2O_4$ after separation from $LiCoO_2$**	$Li_{1.030}CoO_y\,P_{0.025}$	-	-	-	-	-	-	-	-	-	-
**recycled $LiMn_2O_4$ after separation from $LiCoO_2$**	$Li_{1.127}CoO_y\,P_{0.032}$	-	-	-	-	-	-	-	-	-	-
**pristine $LiMn_2O_4$**	$Li_{1.193}Mn_2O_y$	-	-	-	-	-	-	-	-	-	-
**recycled $LiMn_2O_4$ after separation from Li_6PS_5Cl**	$Li_{1.529}Mn_2O_y\,P_{0.001}$	-	-	-	-	-	-	-	-	-	-
**recycled $LiMn_2O_4$ after separation**	$Li_{1.598}Mn_2O_y\,P_{0.041}$	-	-	-	-	-	-	-	-	-	-

**from Li_6PS_5Br**											
**recycled $LiMn_2O_4$ after separation from Li_6PS_5I**	$Li_{1.614}Mn_2O_y P_{0.038}$	-	-	-	-	-	-	-	-	-	-
**pristine NMC811**	$Li_{1.099}Ni_{0.8}Mn_{0.122}Co_{0.112}O_y$	11.2	10.8	77.4	-	-	0.6	-	-	-	$Li_xNi_{0.8}Mn_{0.11}Co_{0.11}O_y S_{0.006}$
		11.0	9.9	78.3	-	-	0.9	-	-	-	$Li_xNi_{0.8}Mn_{0.10}Co_{0.1}O_y S_{0.009}$
		11.1	10.0	78.2	-	-	0.7	-	-	-	$Li_xNi_{0.8}Mn_{0.10}Co_{0.1}O_y S_{0.007}$
**recycled NMC811 after separation from Li_6PS_5Cl**	$Li_{0.991}Ni_{0.8}Mn_{0.121}Co_{0.115}O_y P_{0.011}$	11.0	10.4	77.1	-	0.7	0.8	-	-	-	$Li_xNi_{0.8}Mn_{0.11}Co_{0.11}O P_{0.007}S_{0.008}$
		10.8	10.4	76.7	-	0.8	1.2	-	-	-	$Li_xNi_{0.8}Mn_{0.11}Co_{0.11}O_y P_{0.008}S_{0.012}$
		10.8	10.5	77.1	-	0.7	0.8	-	-	-	$Li_xNi_{0.8}Mn_{0.11}Co_{0.11}O_y P_{0.007}S_{0.008}$
**recycled NMC811 after separation from Li_6PS_5Br**	$Li_{1.005}Ni_{0.8}Mn_{0.114}Co_{0.115}O_y P_{0.014}$	10.2	9.9	72.2	-	3.4	2.1	-	2.3	-	$Li_xNi_{0.8}Mn_{0.11}Co_{0.11}O_y P_{0.037}S_{0.023}Br_{0.025}$
		10.1	9.9	71.6	-	3.9	2.2	-	2.3	-	$Li_xNi_{0.8}Mn_{0.11}Co_{0.11}O_y P_{0.044}S_{0.025}Br_{0.026}$
		10.3	9.9	71.7	-	3.8	2.1	-	2.2	-	$Li_xNi_{0.8}Mn_{0.11}Co_{0.11}O_y P_{0.042}S_{0.023}Br_{0.025}$
**recycled NMC811 after separation from Li_6PS_5I**	$Li_{1.073}Ni_{0.8}Mn_{0.121}Co_{0.116}O_y P_{0.018}$	10.4	9.3	76.3	-	2.1	1.1	-	-	0.8	$Li_xNi_{0.8}Mn_{0.10}Co_{0.11}O_y P_{0.022}S_{0.012}I_{0.008}$
		9.7	9.1	71.2	-	7.3	2.7	-	-	-	$Li_xNi_{0.8}Mn_{0.10}Co_{0.11}O_y P_{0.082}S_{0.030}$
		9.6	8.8	69.5	-	8.6	2.7	-	-	0.8	$Li_xNi_{0.8}Mn_{0.10}Co_{0.11}O_y P_{0.099}S_{0.031}I_{0.009}$
**pristine $LiFePO_4$**	$Li_{0.874}FeP_{0.968}O_y$	-	-	-	46.8	53.2	-	-	-	-	$Li_xFe_1P_{1.14}O_y$
		-	-	-	48.6	51.4	-	-	-	-	$Li_xFe_1P_{1.06}O_y$
		-	-	-	48.4	51.6	-	-	-	-	$Li_xFe_1P_{1.07}O_y$
**recycled $LiFePO_4$ after separation from Li_6PS_5Cl**	$Li_{1.051}FeP_{1.051}O_y$	-	-	-	49.1	50.9	-	-	-	-	$Li_xFe_1P_{1.04}O_y$
		-	-	-	48.0	52.0	-	-	-	-	$Li_xFe_1P_{1.08}O_y$
		-	-	-	48.3	51.7	-	-	-	-	$Li_xFe_1P_{1.07}O_y$
**recycled $LiFePO_4$**	$Li_{1.148}FeP_{1.086}O_y$	-	-	-	48.4	50.0	1.6	-	-	-	$Li_xFe_1P_{1.03}O_y S_{0.033}$
		-	-	-	48.0	50.2	1.8	-	-	-	$Li_xFe_1P_{1.05}O_y S_{0.038}$

**after separation from Li_6PS_5Br**		-	-	-	49.1	49.5	1.4	-	-	-	$Li_xFe_1P_{1.01}O_y$ $S_{0.028}$
**recycled $LiFePO_4$ after separation from Li_6PS_5I**	$Li_{1.188}FeP_{1.122}O_y$	-	-	-	45.2	52.5	1.6	-	-	0.7	$Li_xFe_1P_{1.16}O_y$ $S_{0.036}I_{0.015}$
		-	-	-	46.2	51.8	1.3	-	-	0.7	$Li_xFe_1P_{1.12}O_y$ $S_{0.028}I_{0.015}$
		-	-	-	44.8	52.9	1.5	-	-	0.8	$Li_xFe_1P_{1.18}O_y$ $S_{0.031}I_{0.018}$

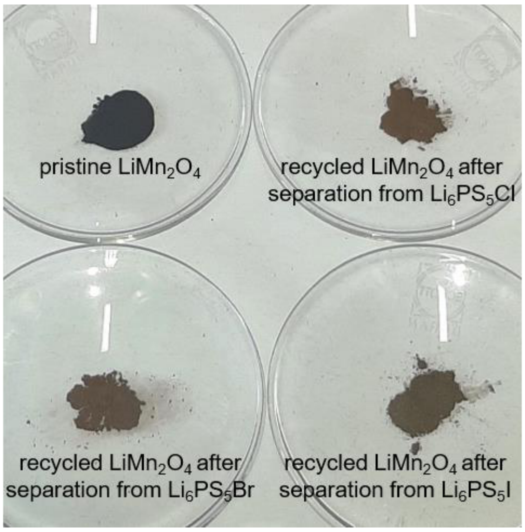

Figure S 9: Photograph of pristine and recycled $LiMn_2O_4$ after solvent treatment in EtOH and separation from Li_6PS_5Cl, Li_6PS_5Br, and Li_6PS_5I, highlighting the color change from black to brown.

Table S 10: Oxidation and reduction peak potentials of pristine and recycled LCO after separation from Li_6PS_5Cl, Li_6PS_5Br, and Li_6PS_5I.

	**Cycle number**	**Oxidation peak potential [V]**				**Reduction peak potential [V]**			
**pristine LCO**	**1st**	4.04		4.20		4.15	4.03	3.84	3.72
	**2nd**	4.05	4.10	4.20		4.15	4.02	3.82	
	**3rd**	4.05	4.10	4.21		4.14	4.01	3.80	
	**4th**	4.08	4.11	4.21		4.13	4.01	3.80	
	**5th**		4.10	4.21		4.13	4.00	3.78	
**recycled LCO after separation from Li_6PS_5Cl**	**1st**	4.06	4.11	4.20		4.14	4.02	3.82	3.65
	**2nd**	4.06	4.11	4.21	4.42	4.13	4.02	3.80	
	**3rd**	4.06	4.10	4.22	4.39	4.13	4.01	3.79	
	**4th**	4.06	4.11	4.22	4.36	4.13	4.01	3.78	
	**5th**	4.05	4.10	4.24	4.39	4.13	4.00	3.77	
**recycled LCO after separation from Li_6PS_5Br**	**1st**	4.08	4.11	4.21		4.14	4.01	3.82	3.65
	**2nd**	4.06	4.10	4.21		4.14	4.01	3.81	
	**3rd**	4.08	4.11	4.21		4.13	4.00	3.78	
	**4th**	4.08	4.12	4.22		4.13	4.00	3.76	
	**5th**	4.09	4.14	4.22		4.12	3.99	3.75	
**recycled LCO after separation from Li_6PS_5I**	**1st**	4.07	4.11	4.20		4.15	4.02	3.82	3.69
	**2nd**	4.08	4.13	4.22		4.14	4.01	3.79	3.69
	**3rd**	4.10	4.14	4.22		4.13	4.00	3.78	3.68
	**4th**	4.12	4.16	4.23		4.12	4.00	3.76	3.65
	**5th**	4.14	4.18	4.25		4.12	3.97	3.75	3.60

Table S 11: Oxidation and reduction peak potentials of pristine and recycled LTO after separation from Li_6PS_5Cl, Li_6PS_5Br, and Li_6PS_5I.

	Cycle number	Oxidation peak potential [V]	Reduction peak potential [V]
**pristine LTO**	**1st**	1.74	1.35
	**2nd**	1.73	1.47
	**3rd**	1.72	1.48
	**4th**	1.71	1.48
	**5th**	1.70	1.49
**recycled LTO after separation from Li_6PS_5Cl**	**1st**	1.70	1.33
	**2nd**	1.71	1.49
	**3rd**	1.72	1.49
	**4th**	1.72	1.48
	**5th**	1.73	1.47
**Recycled LTO after separation from Li_6PS_5Br**	**1st**	1.70	1.35
	**2nd**	1.68	1.51
	**3rd**	1.66	1.51
	**4th**	1.65	1.51
	**5th**	1.64	1.51
**recycled LTO after separation from Li_6PS_5I**	**1st**	1.71	1.34 1.49
	**2nd**	1.70	1.49
	**3rd**	1.70	1.49
	**4th**	1.69	1.50
	**5th**	1.68	1.50

Table S 12: Oxidation and reduction peak potentials of pristine and recycled LMO after separation from Li_6PS_5Cl, Li_6PS_5Br, and Li_6PS_5I.

	Cycle number	Oxidation peak potential [V]				Reduction peak potential [V]		
**pristine LMO**	**1st**			4.15	4.28	4.00	3.84	
	**2nd**			4.13	4.27	3.98	3.84	3.70
	**3rd**			4.13	4.27	3.98	3.84	3.69
	**4th**			4.13	4.28	3.98	3.84	3.69
	**5th**			4.13	4.28	3.98	3.84	
**recycled LMO after separation from Li_6PS_5Cl**	**1st**	3.26	3.83	4.10	4.23	4.01	3.88	
	**2nd**			4.10	4.22	4.01	3.88	
	**3rd**			4.09	4.22	4.02	3.88	
	**4th**			4.09	4.22	4.03	3.88	
	**5th**			4.09	4.21	4.03	3.88	
**recycled LMO after separation from Li_6PS_5Br**	**1st**	3.39	3.89	4.20	4.42	3.99	3.81	
	**2nd**			4.15	4.29	3.98	3.81	
	**3rd**			4.15	4.29	3.98	3.81	
	**4th**			4.14	4.29	3.98	3.82	
	**5th**			4.13	4.28	3.98	3.82	
**recycled LMO after separation from Li_6PS_5I**	**1st**	3.26	3.82	4.11	4.25	4.00	3.85	
	**2nd**			4.10	4.25	4.01	3.85	
	**3rd**			4.09	4.24	4.91	3.86	
	**4th**			4.09	4.24	4.01	3.86	
	**5th**			4.09	4.24	4.02	3.87	

Table S 13: Oxidation and reduction peak potentials of pristine and recycled NMC811 after separation from Li_6PS_5Cl, Li_6PS_5Br, and Li_6PS_5I.

	**Cycle number**	**Oxidation peak potential [V]**					**Reduction peak potential [V]**		
**pristine NMC811**	**1st**			3.93		4.26	4.19	3.96	3.69
	**2nd**		3.81		4.04	4.26	4.18	3.96	3.69
	**3rd**		3.81		4.05	4.27	4.16	3.96	3.68
	**4th**		3.81		4.05	4.27	4.14	3.96	3.68
	**5th**		3.82		4.05	4.27	4.13	3.96	3.68
**recycled NMC811 after separation from Li_6PS_5Cl**	**1st**				4.11	4.31	4.15	3.97	3.67
	**2nd**			3.90		4.32	4.14	3.94	3.66
	**3rd**			3.89		4.32	4.13	3.91	3.65
	**4th**			3.89		4.32	4.11	3.91	3.64
	**5th**			3.89		4.32	4.09	3.90	3.64
**recycled NMC811 after separation from Li_6PS_5Br**	**1st**				4.08	4.35	4.14	3.95	3.65
	**2nd**	3.45		3.92	4.08	4.37	4.11	3.92	3.65
	**3rd**			3.91			4.09	3.92	3.64
	**4th**			3.91			4.06	3.90	3.63
	**5th**			3.92			4.05	3.89	3.61
**recycled NMC811 after separation from Li_6PS_5I**	**1st**			3.96	4.10	4.28	4.16	3.94	3.67
	**2nd**		3.82		4.05	4.27	4.16	3.94	3.66
	**3rd**		3.84		4.07	4.26	4.14	3.96	3.66
	**4th**		3.82		4.06	4.27	4.14	3.97	3.66
	**5th**		3.84		4.06	4.26	4.13	3.96	3.65

Table S 14: Oxidation and reduction peak potentials of pristine LFP.

	**Cycle number**	**Oxidation peak potential [V]**			**Reduction peak potential [V]**
**pristine LFP**	**1st**		3.62	4.12	3.18
	**2nd**	3.34	3.59	4.07	3.18
	**3rd**		3.58	4.02	3.17
	**4th**		3.57	4.02	3.16
	**5th**		3.55	4.02	3.16

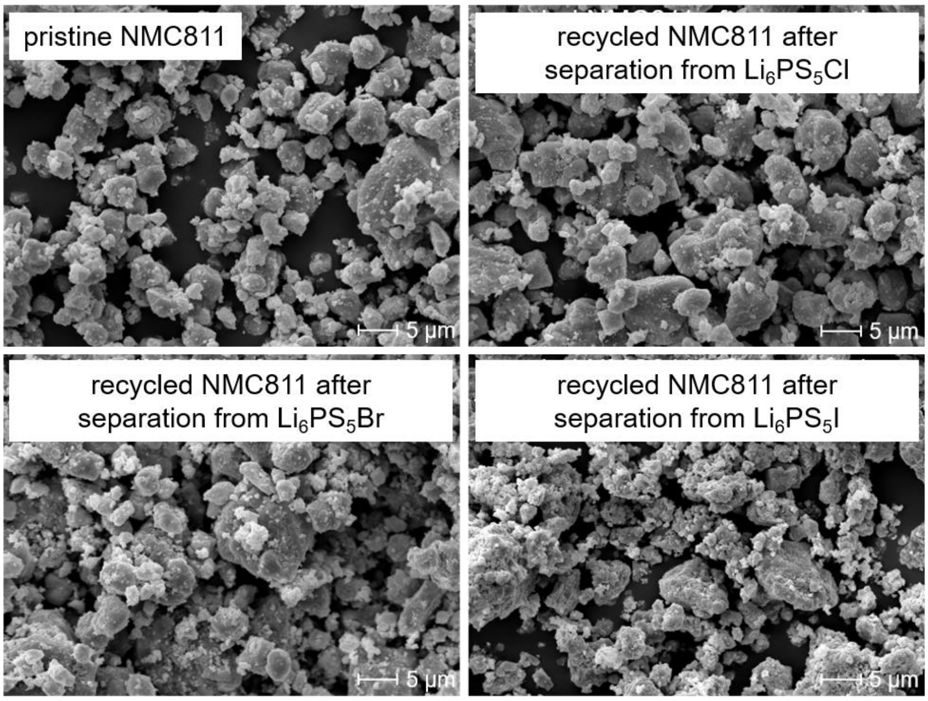

Figure S 10: SE micrographs of pristine and recycled NMC811 after separation from Li_6PS_5Cl, Li_6PS_5Br and Li_6PS_5I. For the dissolution of the SEs, EtOH was used (solid:liquid ratio of 10mg:1ml, stirring time of 24 h).

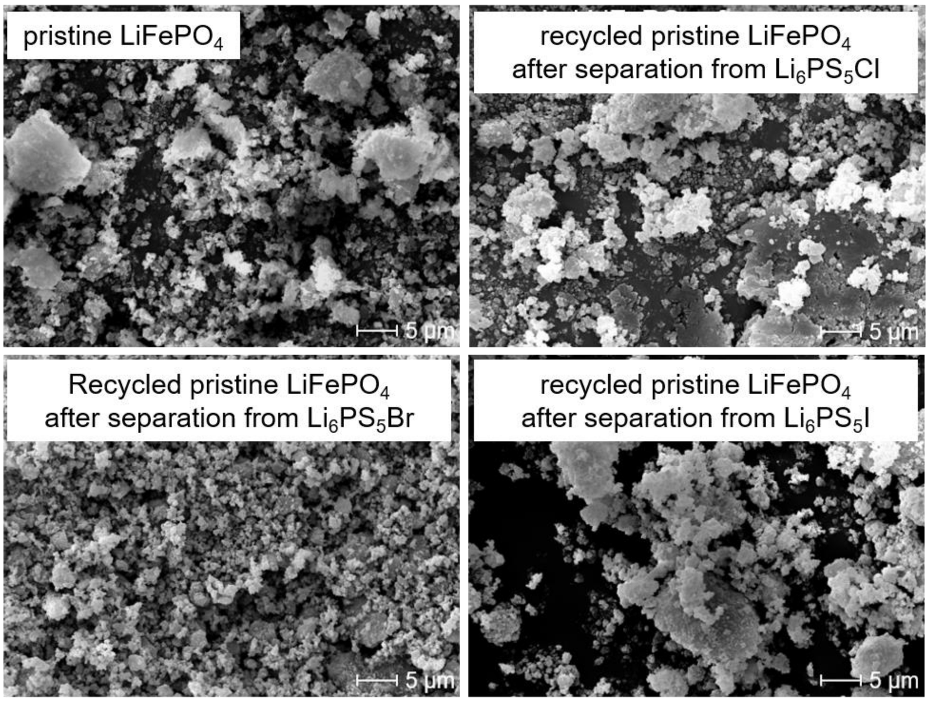

Figure S 11: SE micrographs of pristine and recycled $LiFePO_4$ after separation from Li_6PS_5Cl, Li_6PS_5Br and Li_6PS_5I. For the dissolution of the SEs, EtOH was used (solid:liquid ratio of 10mg:1ml, stirring time of 24 h).

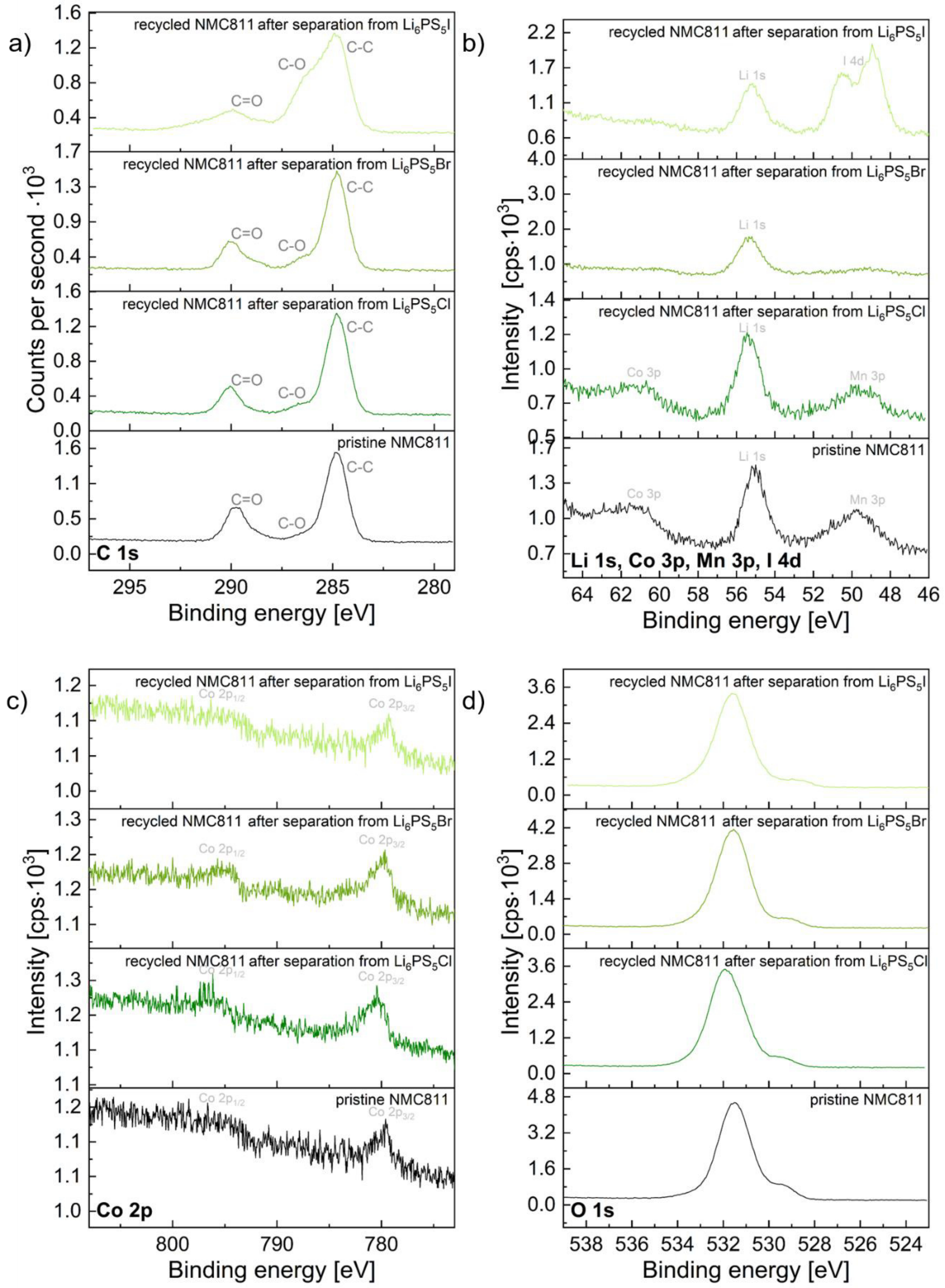

Figure S *12*: C 1s (a), Li 1s/Co 3p/Mn 3p/I 4d (b), Co 2p (c) and O 1s (d) XP spectra of recycled NMC811 after separation from Li_6PS_5Cl, Li_6PS_5Br, and Li_6PS_5I in comparison to pristine NMC811. For the dissolution of the SEs, EtOH was used (solid:liquid ratio of 10 mg:1 ml, stirring time of 24 h).

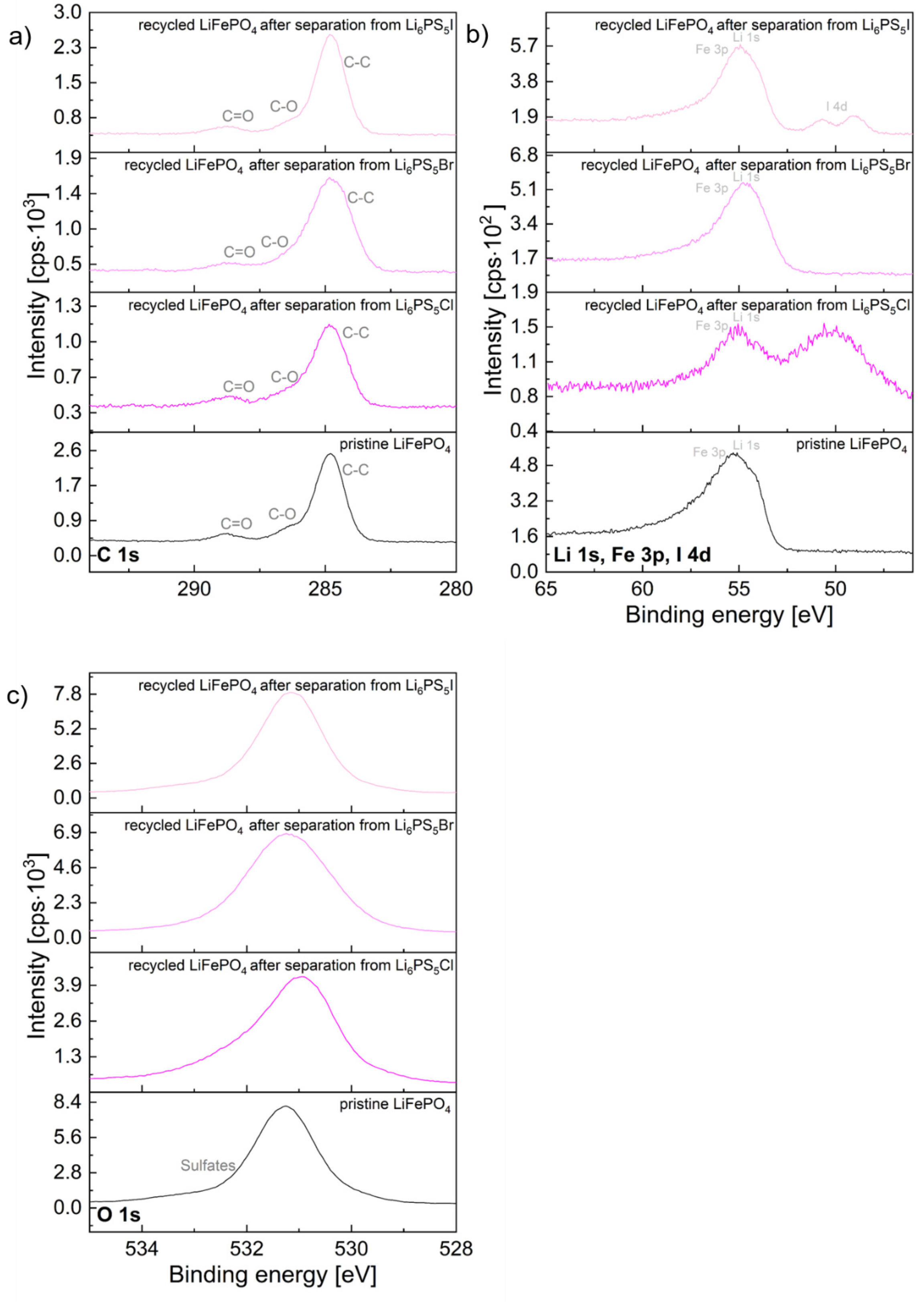

Figure S 13: C 1s (a), Li 1s/Fe 3p/I 4d (b), and O 1s (c) XP spectra of recycled $LiFePO_4$ after separation from Li_6PS_5Cl, Li_6PS_5Br, and Li_6PS_5I in comparison to pristine $LiFePO_4$. For the dissolution of the SEs, EtOH was used (solid:liquid ratio of 10 mg:1 ml, stirring time of 24 h).

References

1. Appendix A. Properties, Purification, and Use of Organic Solvents. In *Solvents and Solvent Effects in Organic Chemistry*, 2010; pp 549-586.
